# A global data-driven census of *Salmonella* small proteins and their potential functions in bacterial virulence

**DOI:** 10.1093/femsml/uqaa002

**Published:** 2020-10-17

**Authors:** Elisa Venturini, Sarah L Svensson, Sandra Maaß, Rick Gelhausen, Florian Eggenhofer, Lei Li, Amy K Cain, Julian Parkhill, Dörte Becher, Rolf Backofen, Lars Barquist, Cynthia M Sharma, Alexander J Westermann, Jörg Vogel

**Affiliations:** Institute of Molecular Infection Biology (IMIB), University of Würzburg, Josef-Schneider-Straße, 97080, Würzburg, Germany; Chair of Molecular Infection Biology II, Institute of Molecular Infection Biology, University of Würzburg, Josef-Schneider-Straße 2, 97080, Würzburg, Germany; Department of Microbial Proteomics, Institute of Microbiology, University of Greifswald, Felix-Hausdorff-Straße, 17489, Greifswald, Germany; Bioinformatics Group, Department of Computer Science, Albert-Ludwigs-University Freiburg, Georges-Köhler-Allee, 79110, Freiburg, Germany; Bioinformatics Group, Department of Computer Science, Albert-Ludwigs-University Freiburg, Georges-Köhler-Allee, 79110, Freiburg, Germany; Institute of Systems and Physical Biology, Shenzhen Bay Laboratory, Guangqiao Road, Guangming District, 518131, Shenzhen, China; Department of Molecular Sciences, Macquarie University, Balaclava Road, Macquarie Park, New South Wales, 2109, Sydney, Australia; Wellcome Trust Sanger Institute, Hinxton, CB10 1SA, Cambridge, UK; Wellcome Trust Sanger Institute, Hinxton, CB10 1SA, Cambridge, UK; Department of Veterinary Medicine, University of Cambridge, Madingley Road, CB3 0ES, Cambridge, UK; Department of Microbial Proteomics, Institute of Microbiology, University of Greifswald, Felix-Hausdorff-Straße, 17489, Greifswald, Germany; Bioinformatics Group, Department of Computer Science, Albert-Ludwigs-University Freiburg, Georges-Köhler-Allee, 79110, Freiburg, Germany; Helmholtz Institute for RNA-based Infection Research (HIRI), Helmholtz Centre for Infection Research (HZI), Josef-Schneider-Straße, 97080, Würzburg, Germany; Faculty of Medicine, University of Würzburg, Josef-Schneider-Straße, 97080, Würzburg, Germany; Chair of Molecular Infection Biology II, Institute of Molecular Infection Biology, University of Würzburg, Josef-Schneider-Straße 2, 97080, Würzburg, Germany; Institute of Molecular Infection Biology (IMIB), University of Würzburg, Josef-Schneider-Straße, 97080, Würzburg, Germany; Helmholtz Institute for RNA-based Infection Research (HIRI), Helmholtz Centre for Infection Research (HZI), Josef-Schneider-Straße, 97080, Würzburg, Germany; Institute of Molecular Infection Biology (IMIB), University of Würzburg, Josef-Schneider-Straße, 97080, Würzburg, Germany; Helmholtz Institute for RNA-based Infection Research (HIRI), Helmholtz Centre for Infection Research (HZI), Josef-Schneider-Straße, 97080, Würzburg, Germany

**Keywords:** small proteins, *Salmonella* Typhimurium, dual RNA-seq, TraDIS, Grad-seq, Ribo-seq, sPepFinder, MgrB, virulence, infection

## Abstract

Small proteins are an emerging class of gene products with diverse roles in bacterial physiology. However, a full understanding of their importance has been hampered by insufficient genome annotations and a lack of comprehensive characterization in microbes other than *Escherichia coli*. We have taken an integrative approach to accelerate the discovery of small proteins and their putative virulence-associated functions in *Salmonella* Typhimurium. We merged the annotated small proteome of *Salmonella* with new small proteins predicted with *in silico* and experimental approaches. We then exploited existing and newly generated global datasets that provide information on small open reading frame expression during infection of epithelial cells (dual RNA-seq), contribution to bacterial fitness inside macrophages (Transposon-directed insertion sequencing), and potential engagement in molecular interactions (Grad-seq). This integrative approach suggested a new role for the small protein MgrB beyond its known function in regulating PhoQ. We demonstrate a virulence and motility defect of a *Salmonella* Δ*mgrB* mutant and reveal an effect of MgrB in regulating the *Salmonella* transcriptome and proteome under infection-relevant conditions. Our study highlights the power of interpreting available ‘omics’ datasets with a focus on small proteins, and may serve as a blueprint for a data integration-based survey of small proteins in diverse bacteria.

## Teaser

This study discovers and evaluates candidate small proteins using new and existing Ribo-seq, dual RNA-seq and TraDIS data, illustrating how data reuse and integration can accelerate determination of gene function.

## INTRODUCTION

Small proteins, loosely defined as shorter than 100 amino acids (aa), are being increasingly implicated in regulating major biological processes in all kingdoms of life (Storz, Wolf and Ramamurthi 2014; Saghatelian and Couso 2015; Makarewich and Olson 2017). In bacteria, several small proteins have long been known to perform both structural and regulatory functions in ribosomal subunits. Another major class is that of small protein members of toxin-antitoxin systems, particularly type-II, where both the toxin and the antitoxin are proteins (Harms *et al*. 2018). However, starting with the discovery almost two decades ago of previously overlooked conserved small open reading frames (sORFs) in the *Escherichia coli* chromosome (Wassarman *et al*. 2001), interest in other potential roles of bacterial small proteins has been increasing (Storz, Wolf and Ramamurthi 2014; Miravet-Verde *et al*. 2019; Sberro *et al*. 2019). In-depth characterization of individual small proteins has since revealed an unexpected diversity of functions in several different species. For example, the *E. coli* small protein MgtS (31 aa) indirectly increases the intracellular level of magnesium by binding and regulating the activity of the magnesium importer MgtA (Wang *et al*. 2017) and the cation-phosphate symporter PitA (Yin *et al*. 2019). SgrT (43 aa), a small protein encoded by the dual-function small RNA (sRNA) SgrS in Enterobacteriaceae, inhibits the activity of the major glucose transporter PtsG under sugar-phosphate stress (Lloyd *et al*. 2017). In *Listeria monocytogenes*, Prli42 (31 aa) is essential for survival in macrophages as a previously overlooked member of the stressosome (Impens *et al*. 2017). The variety of functions that have so far been attributed to the few characterized small proteins suggests that much remains to be discovered. Moreover, a clear picture of how many *bona fide*, translated sORFs are encoded even by otherwise well-studied model bacteria is currently lacking.

While the prevalence and functionality of bacterial small proteins remain best understood in the non-pathogenic *E. coli* strain K12 (Hemm, Weaver and Storz 2020), there is increasing evidence for small protein functions in related enteric pathogenic bacteria, especially in *Salmonella enterica* serovar Typhimurium (henceforth, *Salmonella*). Pre-genomic work showed that these two model species of microbiology differ by several large genetic regions that *Salmonella* acquired in its evolution towards becoming an intracellular pathogen of eukaryotic hosts (Groisman and Ochman 1996). For example, the *Salmonella*pathogenicity islands 1 and 2 (SPI-1, SPI-2) each encode a type-III secretion system (T3SS) that translocates its corresponding effector proteins into the host cell where they modulate host cellular processes to the bacterium's benefit (Patel and Galán 2005; Jennings, Thurston and Holden 2017). However, early genomic comparisons showed that the large majority of genetic differences between the *E. coli* and *Salmonella* genomes are small (Parkhill *et al*. 2001), while there are numerous distinctive regions in the *Salmonella* genome that encode different virulence-associated factors (Dos Santos, Ferrari and Conte-Junior 2019). These loci might harbour previously overlooked small proteins. In other words, a systematic annotation and analysis of small proteins in *Salmonella* promises not only to unveil functions of *E. coli* sORFs that are conserved (or deviate) in a related species, but it might also reveal *Salmonella*-specific small proteins, some of which might contribute to virulence of this pathogen.

Indeed, while only a handful of small proteins have been characterized in *Salmonella*, several of them proved to have a virulence-related function. For example, we recently found the cold-shock proteins CspC and CspE (69 and 70 aa, respectively) to be essential for *Salmonella* pathogenicity and demonstrated a global RNA-binding function for these two small proteins (Michaux *et al*. 2017). Besides these examples, the small protein MgtR (30 aa) promotes the degradation of MgtC, thereby contributing to titration of the levels of this virulence factor (Alix and Blanc-Potard 2008). At the same time, together with MgtS (see above), MgtR regulates the proteolysis of the magnesium importer MgtA under Mg^2+^ limiting conditions that *Salmonella* typically experiences inside its host cell (Choi, Lee and Shin 2012). MgtR also regulates the degradation of another magnesium transporter, MgtB, while another novel small protein from the same locus as MgtR, MgtU, independently stabilizes MgtB by interfering with its FtsH-mediated degradation (Yeom, Shao and Groisman 2020). Also related to infection, PmrD (85 aa) is required for the response of the PmrAB two-component system (TCS) that confers resistance to antimicrobial peptides (Kox, Wösten and Groisman 2000).

The MgrB small protein (47 aa) has been of special interest because it binds and inhibits the PhoQ kinase of the PhoPQ TCS, which is the central regulator of *Salmonella*’s intracellular virulence program (Lippa and Goulian 2009). The inhibition of PhoPQ is conserved in *E. coli*, where MgrB was shown to localize to the cell membrane, and where this small protein inhibits the kinase activity of PhoQ as part of a negative feedback loop (Salazar, Podgornaia and Laub 2016), since *mgrB* transcription itself is activated through PhoPQ (Kato, Tanabe and Utsumi 1999). Recently, interest in MgrB has further increased as several reports linked this small protein to antibiotic resistance development in the opportunistic pathogen *Klebsiella pneumoniae* (Poirel *et al*. 2015; Zowawi *et al*. 2015; Kidd *et al*. 2017). These case studies notwithstanding, our knowledge of the role of MgrB in bacterial virulence, and the potential contribution of other small proteins to *Salmonella* infections, remains poorly understood.

**Figure 1.**
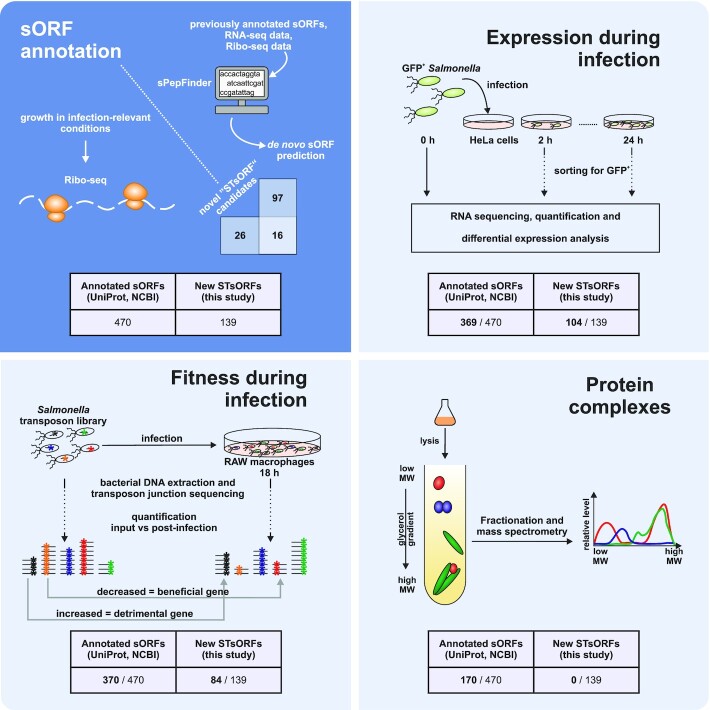
Overview of the *in silico* and experimental approaches included in the present study. For the prediction of novel small proteins (top left), we included results from sPepFinder, a pipeline for the *de novo* prediction of sORFs in bacterial genomes, as well as data from ribosome profiling performed on *Salmonella* grown in infection-relevant conditions. To prioritize candidates for functional characterization, we explored data derived from dual RNA-seq, transposon-directed insertion sequencing (TraDIS), and gradient profiling (Grad-seq). Dual RNA-seq (top right) shows the expression pattern of each gene during infection thanks to the enrichment of host cells infected with GFP-expressing *Salmonella*. Total RNA from these cells is sequenced and the bacterial transcriptome is analysed, calculating the abundance of each transcript relative to the bacterial inoculum (0 h). TraDIS (bottom left) informs on sORFs whose disruption by transposon insertion affects fitness during infection. Gradient profiling (or Grad-seq, bottom right) makes use of a linear glycerol gradient to separate the soluble complexes in *Salmonella* lysates based on shape and molecular weight. Mass spectrometry analysis of the gradient fractions shows the sedimentation pattern of each protein, and correlation of the distribution profiles of individual proteins provides information about their potential interactome. The numbers reported below each cartoon show the small proteins detected with the respective technique.

To close this knowledge gap, we set out to systematically study the virulence-related small proteome of *Salmonella*. We employed both computational predictions (sPepFinder; (Li and Chao 2020)) and experimental ribosome profiling data to further expand the sORF annotation of *Salmonella*. In a second step, given that numerous global datasets exist for this model pathogen, we re-analysed some of these with a focus on our updated small protein annotation (Fig. 1). Specifically, we revisited available data that provide information on gene expression during host cell infection (dual RNA-seq; (Westermann *et al*. 2016)), on *Salmonella* mutant fitness upon uptake by macrophages (Transposon-directed insertion sequencing [TraDIS] (Cain *et al*. 2020)), as well as gradient profiling by mass spectrometry (Grad-seq) data (Smirnov *et al*. 2016) that indicate a possible involvement of the small proteins in cytosolic complexes. We also discuss the strengths and weaknesses of the individual approaches in the context of sORF characterization. This integrative reanalysis provides a starting point for identifying small proteins with virulence factor potential. In particular, given the appearance of the small protein MgrB in several datasets, we set out to characterize its role in *Salmonella* virulence. We report a requirement of MgrB for efficient infection of both epithelial cells and macrophages and describe the impact of this conserved small protein on the *Salmonella* transcriptome and proteome in infection-relevant conditions. MgrB may serve to illustrate how re-inspection of existing omics datasets can help to prioritize candidate small proteins for functional characterization in an organism of interest.

## RESULTS

### Biocomputational search for novel sORF candidates in*Salmonella*

The *Salmonella* SL1344 genome encodes 4657 annotated proteins (as of April 2019), of which 470 are shorter than 100 aa. This category is underrepresented compared to average-sized ORFs (100–500 aa long; Fig. S1), which represent the vast majority of the coding sequences (CDSs). The proteins length distribution of annotated genes is similar to that of the model organism *E. coli* MG1655, to which new small proteins have been successfully added (Weaver *et al*. 2019; Hemm, Weaver and Storz 2020).

To further expand the sORF annotation in *Salmonella*, we searched for previously overlooked small protein candidates by combining computational sORF predictions with experimental data. To this end, we used the predictions generated from the recently developed sPepFinder (Li and Chao 2020), a machine learning-based tool for sORF annotation in bacterial genomes. With the ability to train on known examples and subsequently identify subtle, unrecognized features of a true CDS, a machine-learning-based tool can help to discern real sORFs from false-positive in-frame start and stop codons that do not encode a protein. Briefly, sPepFinder uses a support vector machine (SVM) and 29 features, including a thermodynamic model of ribosome binding sites and the frequency of hydrophobic amino acids, extracted from a training set of annotated bacterial small proteins from ten species, including *Salmonella*.

sPepFinder predicted 340 candidate sORFs with a length cut-off of 100 codons (Table S1). We filtered these to exclude candidates that did not pass the statistical filtering cut-off (SVM probability > 0.9). Furthermore, since sPepFinder predictions are based solely on genomic features, we sought to obtain independent evidence that the identified sORF candidates are indeed expressed. To this end, we interrogated the SalComMac database (Srikumar *et al*. 2015), which contains transcriptomic data of *Salmonella* grown in diverse conditions, including those related to pathogenesis (e.g. during growth inside macrophages). Applying these filters reduced the number to 113 potential new sORFs. For these newly predicted genes, we propose the nomenclature ‘STsORF’ followed by sequential numbers, based on their position in the genome.

Some examples of candidates with condition-specific expression patterns include the candidate STsORF7 (31 aa), induced upon low pH shock, unlike the downstream STsORF8 (Fig. S2a). A substantial number of sORF candidates were expressed under infection-related conditions, namely when *Salmonella* was grown under an invasion gene-inducing condition (referred to as ‘SPI-1’ condition or late exponential phase; exemplified by STsORF62; 47 aa), or in minimal medium mimicking the intravacuolar environment (‘SPI-2 low Mg^2+^’; e.g. STsORF37; 18 aa) (Fig. S2b). Supported by both *in silico* genome-based predictions and available transcriptomic data, we added these 113 novel sORF candidates to the *Salmonella* gene annotation.

### Experimental prediction of *Salmonella* sORFs using Ribo-seq

To further validate the sPepFinder candidates and discover additional sORFs based on association of their mRNAs with translating ribosomes, we used the genome-wide experimental approach of ribosome profiling by sequencing (Ribo-seq; (Ingolia *et al*. 2009; Ingolia *et al*. 2012)). By sequencing the ∼30 nt mRNA fragments that are protected from nuclease digestion by actively translating ribosomes, Ribo-seq provides a global picture of the transcripts being translated at a given time, enabling the determination of ORF boundaries, and has already successfully been applied to refine bacterial sORF annotations (Weaver *et al*. 2019; Canestrari *et al*. 2020). Ribo-seq overcomes some of the limitations of sORF identification based on mass spectrometry approaches, namely dependence on amino acid reference sequences and the detection of multiple peptides per protein. This notwithstanding, Ribo-seq may yield false-positive sORF candidates, e.g. due to enrichment of abundant transcripts that are protected from degradation or non-coding ribosome-binding transcripts (Fremin and Bhatt 2020). Moreover, due to technical limitations, bacterial Ribo-seq data lack a 3-nucleotide periodicity in ORF coverage, which can hamper sORF annotation. Thus, we advocate for the independent validation of a subset of the Ribo-seq-derived candidates by epitope tagging and immunoblotting to enhance the robustness of the genome-wide prediction.

**Figure 2.**
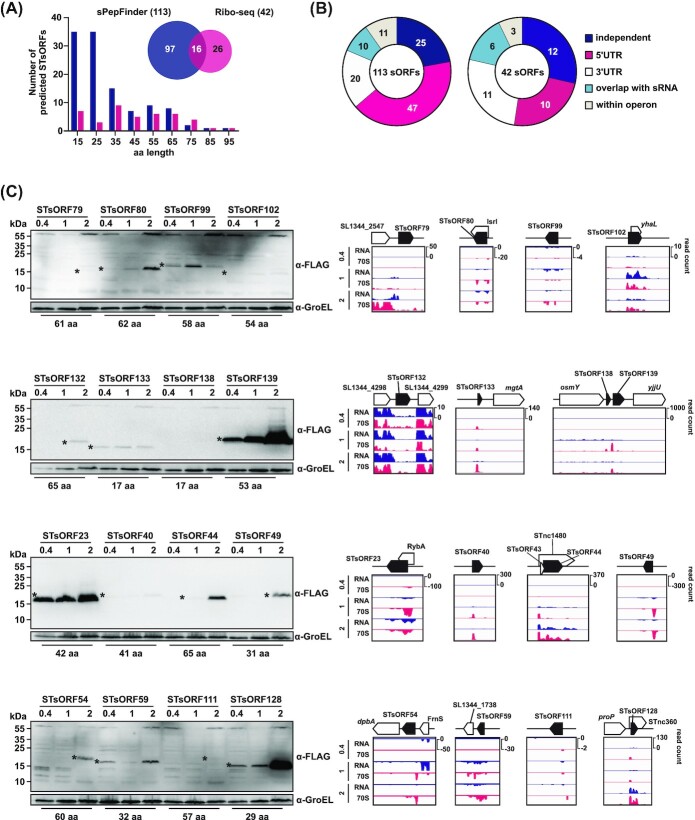
STsORF predictions and validations. **A**, Overlap (Venn diagram) and length distribution (histogram) of the new small proteins identified with sPepFinder and Ribo-seq data. **B**, Genomic distribution of the new STsORFs relative to currently annotated genes as predicted by sPepFinder (left) and Ribo-seq (right). **C**, Validation of the sixteen candidates common to both sPepFinder and Ribo-seq. The STsORFs were tagged at their C-terminus with a SPA-tag, grown in three conditions (LB to an OD_600_ of 0.4, SPI-1- and SPI-2-inducing, as indicated above each lane with ‘0.4’, ‘1’ and ‘2’), and analysed via western blotting. For each sample, 0.1 total ODs were loaded in each lane. Asterisks indicate the small protein bands. GroEL was probed as a loading control. STsORF138 could not be detected on western blots under the examined conditions. On the right side of each western blot, the coverage plots (RPKM values; ‘RNA’ = total RNA, ‘70S’ = ribosome footprints) from Ribo-seq performed on cells grown in the same conditions as for western blotting are shown.

Here, we applied Ribo-seq to *Salmonella* grown under three different *in vitro* conditions (mid-exponential phase in Lennox-Broth (LB) (OD_600_ of 0.4), SPI-1-inducing, or SPI-2-inducing). The resulting data were analysed with REPARATION (Ndah *et al*. 2017), a tool for bacterial gene annotation based on Ribo-seq data. This resulted in the identification of 356 known sORFs and 282 previously unannotated sORF candidates (≤100 aa; Table S1). Application of an additional abundance filter for the total RNA (see Methods) and visual inspection of read coverage profiles (see Methods) produced a final shortlist of 42 sORFs. These overlap with 16 sPepFinder sORFs candidates, adding up to 139 new high-confidence sORFs (Fig. 1) mostly shorter than 50 aa (Fig. 2A). Comparison with published *Salmonella* TSS annotations (Kröger *et al*. 2013) revealed an enrichment of 5’UTR- and independently-encoded sORFs among our new candidates (Fig. 2B).

### Validation of new sORF candidates

The overlap between sORF candidates predicted by both Ribo-seq and sPepFinder was small (16 out of 139; Fig. 2A). This could partially be due to the fact that sPepFinder was trained only on previously annotated small proteins, whose properties may not necessarily be representative of the full complement of bacterial sORFs. Ribo-seq, on the other hand, was performed on cells grown in just three experimental conditions, which has the risk of missing conditionally translated small proteins. Given the complementary nature of the two screens, and our stringent curation parameters, we have included the full set of 139 STsORFs in our updated *Salmonella* small protein annotation, bringing the total small proteome to 609 entries.

To add another level of confidence to these global analyses, we selected the 16 new STsORFs that were predicted by both sPepFinder and Ribo-seq for independent validation by western blot. The respective sORFs were chromosomally tagged at their C-terminus with the sequential peptide affinity (SPA) tag (Zeghouf *et al*. 2004), an epitope previously used to detect small bacterial proteins by immunoblotting (Baek *et al*. 2017; Weaver *et al*. 2019). Protein samples from each tagged strain grown in LB medium to an OD_600_ of 0.4, as well as under SPI-1- or SPI-2-inducing conditions, were collected and analysed by western blot. In this way, we confirmed the translation of 15 candidates (Fig. 2C), mostly highly conserved within *Salmonella* species (exceptions are shown in Fig. S3).

### sORF expression kinetics during host cell infection

**Figure 3.**
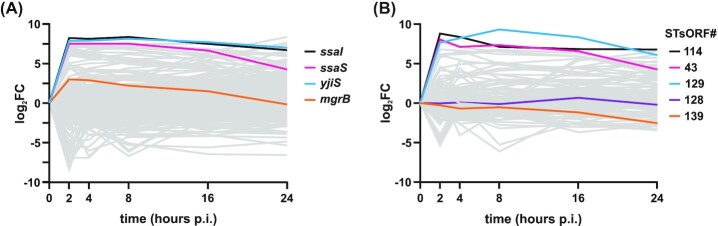
Small protein expression patterns during infection. **A**, Expression of 280 annotated sORFs detected in dual RNA-seq data and significantly regulated (FDR < 0.05) in comparison to the inoculum (0 h). The highlighted transcripts are the top three most highly induced sORFs (log_2_FC > 7 at 2 h p.i.), as well as *mgrB* (log_2_FC = 3 at 2 h). **B**, Expression of 101 new STsORFs detected as significantly regulated with respect to the inoculum (FDR < 0.05) over the course of infection. The highlighted transcripts are the top three most highly induced STsORFs (STsORF114, STsORF43 and STsORF129) and the validated candidates STsORF128 and STsORF139.

Induction of sORF transcription during infection may be an indication of a virulence-related function of the corresponding small protein. Multiple novel and previously annotated sORFs were highly expressed under infection-related conditions, both in SalComMac (Srikumar *et al*. 2015) and in our Ribo-seq data (Fig. 2). To further pinpoint small proteins with a likely role in virulence, we re-analysed global gene expression of intracellular *Salmonella* during epithelial cell infection (Westermann *et al*. 2016) with our updated sORF annotation. This revealed that 280 out of 470 annotated sORFs were significantly differentially expressed throughout infection (false discovery rate [FDR] < 0.05) compared to their expression levels in the inoculum (Fig. 3A, Table S2). The three most highly induced known sORFs at an early infection stage (2 h) encode members of the T3SS apparatus (SsaS and SsaI; 88 and 82 aa, respectively) and the uncharacterized protein YjiS (54 aa). Additionally, expression of *mgrB* (a.k.a. *yobG* in *Salmonella* (Lippa and Goulian 2009)) peaked at 2 h post-infection (p.i.), but remained elevated up to 16 h p.i.

Furthermore, we detected expression of 104 new STsORFs, 101 of which were significantly (FDR < 0.05) regulated throughout infection compared to the inoculum (Fig. 3B). The three most highly induced representatives were STsORF114, STsORF43, and STsORF129. STsORF114 (16 aa), encoded in the 5’UTR of *mgtC* (Fig. S4a), mimics the expression pattern of *mgtC*, encoding a known *Salmonella* virulence factor (Lee and Lee 2015). Further analysis indicated that STsORF114 is a homolog of the *Salmonella* 14028s protein MgtP, one of the two characterized small proteins encoded in the 5’UTR of *mgtC* that regulate its transcription (Lee and Groisman 2012), and is so far not annotated in the strain SL1344. STsORF43 (13 aa) is encoded together with STsORF44 (65 aa; Fig.   2C) within the sRNA STnc1480 (Fig. S4a), whose expression is PhoP- and SlyA-dependent (Colgan *et al*. 2016). STsORF129 (32 aa) is encoded downstream of—and possibly expressed from an annotated TSS internal to—the acid phosphatase gene *phoN* (Fig. S4b). These three highly-induced STsORFs did not result in a tBLASTn hit in bacterial species outside *Salmonella*, further pointing towards a *Salmonella*-specific role in infection (Fig. S4a, b). To validate induction of the corresponding small proteins under SPI-2-inducing conditions, we tagged their respective CDSs with a SPA-tag at their C-terminus and performed western blot analysis. This confirmed the translation of STsORF114 and STsORF43 under conditions mimicking the vacuolar compartment (Fig. S4c), whereas no signal was obtained for STsORF129. Conversely, the mRNAs of STsORF128 (29 aa) and STsORF139 (53 aa)—encoding two of the novel small proteins that were induced under SPI-2 conditions in the above western blot (Fig. 2C)—were not strongly upregulated inside epithelial cells (Fig. 3B). The latter examples could be due to the defined *in vitro* conditions not being able to fully reconstitute the complex intracellular environment, as is the case for other *Salmonella* genes (Srikumar *et al*. 2015).

### Infection phenotypes of sORF disruption mutants

To further narrow our focus on sORFs with potential functions in virulence, we generated TraDIS data from *Salmonella* infection of macrophages to identify genes whose disruption affected *Salmonella* fitness during infection. This approach has already proven useful to assay gene essentiality of *Salmonella* grown *in vitro* (Barquist *et al*. 2013). In the present work, the composition of a transposon mutant pool 20 h after uptake by murine RAW264.7 macrophages was compared to its composition in the inoculum (Table S2). For each gene targeted by transposon insertion, a positive fold-change indicates that the given mutant was over-represented in the pool after infection compared to the input, suggesting the loss of the protein to be beneficial for virulence. A negative fold-change instead indicates that the corresponding protein might be required for infection.

As expected, mutants of the *rfa/rfb* clusters, involved in lipopolysaccharide O-antigen assembly, were strongly enriched after infection (log_2_FC up to 5.8), in line with previous findings (Zenk, Jantsch and Hensel 2009). Conversely, mutants of SPI-2 genes were depleted from the pool (e.g. *ssaV* and *sseC*, both with a log_2_FC = -1.2) along with purine biosynthesis genes (e.g. *purE*, log_2_FC = −4.1), in accordance with their known requirement for intracellular survival (Fields *et al*. 1986; Klein and Jones 2001; Browne *et al*. 2008) and consistent with a recent TraDIS study of *Salmonella* ST313 strain D23580 gene requirements for infection of RAW264.7 macrophages (Canals *et al*. 2019).

**Figure 4.**
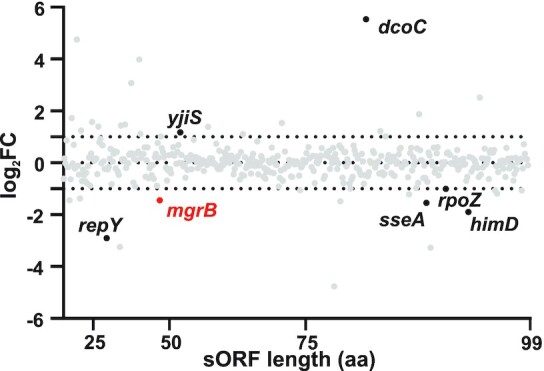
Requirement of small proteins during infection. Plot of the 454 sORFs shorter than 100 aa with transposon insertions detected in the TraDIS pool. The y-axis displays the log_2_FC of each mutant after quantification in the starting pool vs 20 h p.i. of RAW264.7 macrophages (data were collected from two biological replicates). The genes are distributed along the x-axis based on the length (aa). The seven sORFs highlighted are the ones with |log_2_FC| > 1 and q-value < 0.05.

Even though random mutagenesis is inherently biased against small genes, our transposon library (∼ 100 000 insertions) included mutants for 454 of the 609 *Salmonella* sORFs. The insertion rate ranged between 1 and 30 per gene (Fig. S5), with four exceptions having between 80 and 125 insertions, all encoded on the *Salmonella* plasmid pRSF1010. We filtered our sORFs for an infection phenotype based on two criteria, namely significance (q-value < 0.05) and fold-change in relative abundance (|log_2_FC| > 1) (Fig. 4, Table S2). None of the novel STsORFs that were disrupted by a transposon (84) passed the statistical filtering cut-off, despite having log_2_FC values ranging from 4.7 to −3. Among the previously annotated sORFs, seven passed the filtering criteria for an infection phenotype (q-value < 0.05 and |log_2_FC| > 1, Fig. 4, Table S2). For example, disruption of *sseA* (encoding a structural protein of the SPI-2 T3SS; 89 aa), *himD* (encoding the β subunit of integration host factor, IHF; 94 aa), *rpoZ* (encoding a subunit of the RNA polymerase, RNAP; 91 aa), *repY* (encoding the regulator of the pCol1B9 plasmid replication initiation protein RepZ; 29 aa) and *mgrB* (47 aa; also induced inside epithelial cells, Fig. 3A) attenuated infection. In contrast, disruption of *yjiS* (54 aa), which is one of the most highly induced *Salmonella* sORFs inside epithelial cells (Fig. 3A), led to a hyper-virulent phenotype. Similarly, mutants with transposon insertions in *dcoC* (annotated as the gene encoding a putative 81 aa long oxaloacetate decarboxylase) were enriched after infection.

What could be the potential impact of these small proteins on infections? While loss of RpoZ is likely to affect RNAP function and hence bacterial fitness in general, interference with the SPI-2 secretion apparatus in the case of SseA disruption is expected to compromise intracellular survival. HimD is a similar case, as a fully assembled IHF is required for efficient expression of virulence genes (Mangan *et al*. 2006) and for *Salmonella* survival at late stages of macrophage infection (Yoon *et al*. 2009). In contrast, the hypervirulence phenotype of the uncharacterized sORF *yjiS*, particularly in combination with its intracellular induction, implicates this small protein as a novel factor involved in repressing *Salmonella* virulence. In macrophage TraDIS data previously obtained for *S*. Typhimurium ST313 strain D23580 (Canals *et al*. 2019), mutants in the sORF *rpoZ* were impaired at infection and the fold-changes for *sseA*, *himD* and *mgrB* were consistent in direction with ours but not significant (Canals *et al*. 2019). This could reflect both experimental and biological differences between the two studies (e.g. 12 h of growth for D23580 vs. 20 h for SL1344, different analysis tools and genetic differences between both strains).

### Small proteins engaged in larger cellular complexes

Several bacterial small proteins have been found to be integral parts of protein complexes in both the cytosol and the membrane (Storz, Wolf and Ramamurthi 2014). To systematically identify molecular interaction partners of *Salmonella* small proteins, we turned to another dataset that provides a global overview of intracellular (ribonucleo-)protein complexes. The Grad-seq approach relies on the separation of soluble cellular complexes on a linear glycerol gradient according to their size and shape, followed by parallel mass spectrometry of each fraction (Smirnov *et al*. 2016). This provides insights into the cellular complexes a given protein might be engaged in. A small protein not interacting with any other cellular macromolecule would be expected to localize to the low-density fractions of the gradient. In contrast, the localization of a protein in a higher density fraction is indicative of this protein being part of a larger macromolecular complex in the bacterial cell. Of note, while dual RNA-seq and Ribo-seq provide evidence for transcription and translation of a given sORF under a given condition, and TraDIS provides genome-wide genetic evidence for an effect on bacterial fitness during infection, none of these approaches operates at the protein level. In contrast, Grad-seq is coupled to mass spectrometry, and thus allows for the detection of the actual gene products of sORFs.

**Figure 5.**
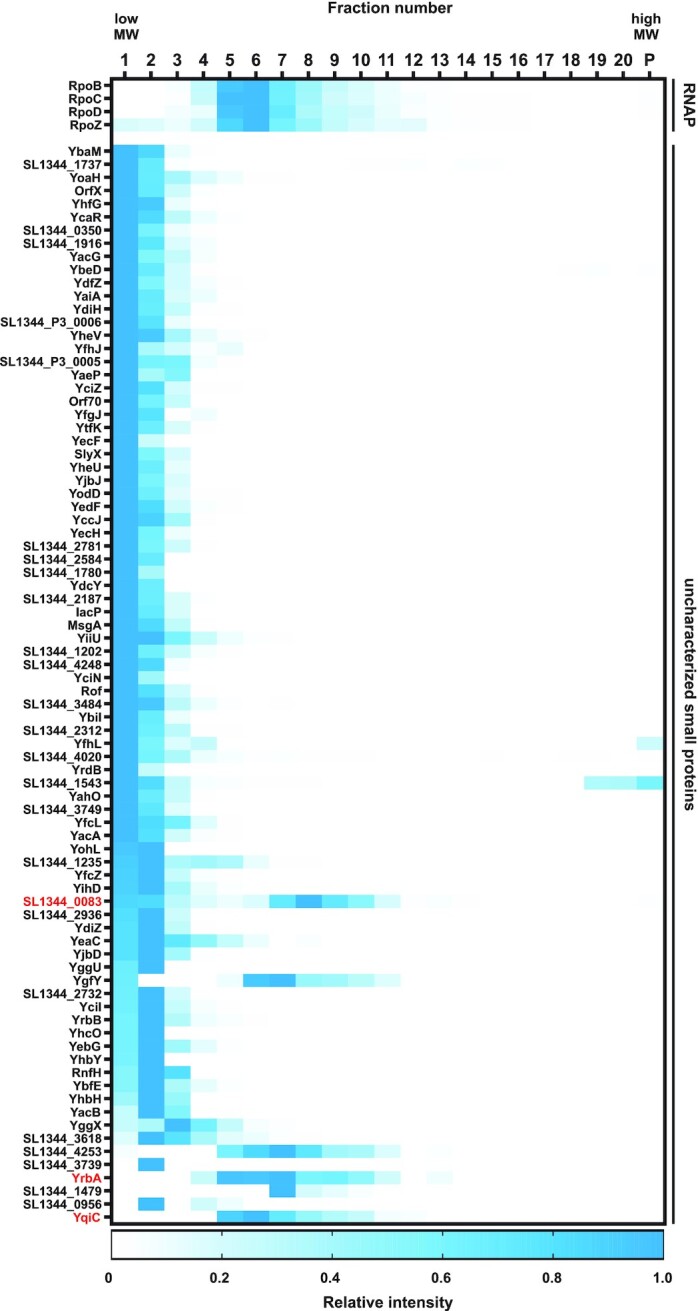
Sedimentation profiles of soluble small proteins. Heat map showing the sedimentation profiles of proteins that are part of RNAP and of the 82 uncharacterized small proteins detected in Grad-seq under SPI-1-inducing conditions that localize beyond the first fraction. The intensity in each fraction, including the pellet (‘P’), was normalized relative to the fraction with the highest intensity for each protein. The proteins in red are mentioned throughout the text.

In total, 170 of our 609 small proteins were detected in the Grad-seq dataset from *Salmonella* grown in SPI-1-inducing conditions (Table S2). These include all 22 annotated small ribosomal proteins, three RNA-binding proteins (CsrA, CspC and CspE), as well as 89 uncharacterized proteins for which no function has been annotated, providing direct evidence for their translation. As a proof of principle for the validity of Grad-seq-derived information on small proteins, the levels of the small RNAP subunit RpoZ (∼10 kDa, 91 aa) peaked in fraction six, the same fraction where the ∼450 kDa RNAP holoenzyme (RpoB-D; Fig. 5) migrates. In contrast, none of the novel STsORFs added to the protein list were detected in this dataset. This is expected for the sPepFinder-derived sORFs, since the tool annotates genes irrespective of the condition they might be expressed in and thus might not be expressed under growth conditions examined by Grad-seq. Another obstacle is that mass spectrometry is limited in the detection of small proteins, since they give rise to fewer unique peptides, as well as lowly abundant and hydrophobic proteins. Since we required at least two peptides for a protein to be considered, it is possible that some of our candidates were lost due to low sensitivity. Indeed, the length distribution of our STsORFs is on average shorter than the ones already annotated (Fig. 2A, Fig. S1).

Among the detected 89 uncharacterized small proteins, seven were only present in the first fraction, suggesting that they are not engaged in stable molecular interactions in the given experimental condition. The sedimentation profiles of the remaining 82 uncharacterized proteins (Fig. 5) showed a variety of patterns. For example, YqiC and YrbA both peaked around fraction six, where the RNAP also sediments. Their homologues present in *E. coli*, YqiC and IbaG, respectively, are most abundant in the second fraction (Hör *et al*. 2020a), suggesting a divergent role between the two organisms. Two peaks were detected for the hypothetical protein SL1344_0083 (one in the low molecular weight fractions and one partially overlapping that of RNAP), suggesting that only a fraction of the SL1344_0083 proteins in a cell are engaged in stable interactions under the analysed condition. Unlike the previous examples, SL1344_0083 is not conserved in *E. coli*. The virulence-related small proteins MgrB and YjiS were absent from the Grad-seq dataset, probably for different reasons. While YjiS is not expressed in the growth condition used for Grad-seq (Fig. 3A), MgrB is an inner membrane-associated protein and the lysis approach used here does not efficiently recover hydrophobic proteins.

In summary, careful analysis of the above global datasets with focus on our refined sORF annotation pinpointed novel infection-relevant small protein candidates (Fig. S6). These include both previously annotated proteins such as YjiS and MgrB, as well as several novel sORFs (STsORF114, STsORF43 and STsORF129).

### *Salmonella* MgrB contributes to macrophage and epithelial cell infection

**Figure 6.**
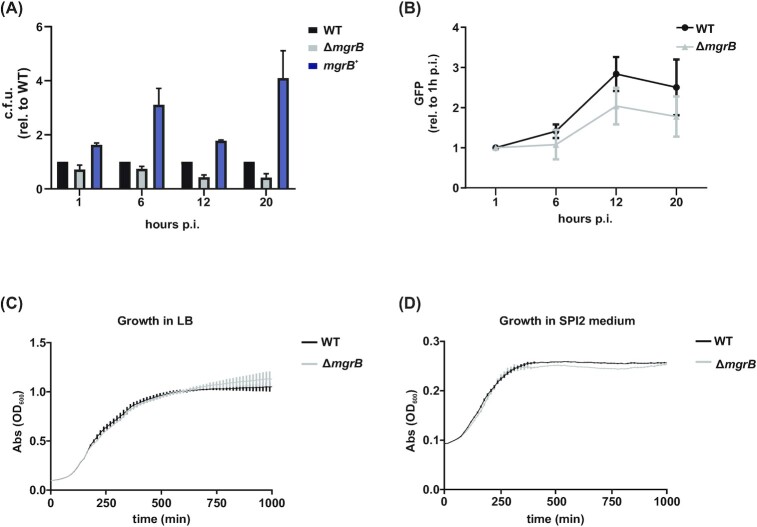
The requirement of MgrB for infection. **A**, Gentamicin protection assay of wild-type or Δ*mgrB Salmonella* carrying the empty vector control, as well as Δ*mgrB* carrying *mgrB* on a plasmid expressed from its native promoter (*mgrB^+^*), infecting RAW264.7 macrophages. C.f.u. counts at each time point were normalized to the wild-type strain. **B**, Intracellular replication in RAW264.7 macrophages of *Salmonella* wild-type or Δ*mgrB* expressing GFP. Fluorescence was normalized to the 1 h time point. For **A** and **B**, data were collected from three biological replicates with error bars indicating standard deviations from the mean. **C**, Growth curve of *Salmonella* wild-type and Δ*mgrB* in LB and SPI-2 medium. For both graphs, data were collected from three biological replicates and bars represent standard deviations from the mean.

The regulation that MgrB exerts on the sensor kinase PhoQ has been described in both *Salmonella* and *E. coli* (Lippa and Goulian 2009). By localizing to the membrane and interacting with PhoQ, MgrB blocks the phosphorylation and subsequent activation of PhoP, inhibiting expression of PhoPQ target genes including *mgrB* itself (Kato, Tanabe and Utsumi 1999; Salazar, Podgornaia and Laub 2016). In our datasets, we observed that MgrB was among the small proteins whose mRNA was induced during infection of epithelial cells (dual RNA-seq; log_2_FC = 3 at 2 h p.i.) and whose inactivation attenuated *Salmonella* infection in murine macrophages (TraDIS; log_2_FC = −1.44). Considering this, we examined the role of MgrB in *Salmonella* virulence. Independent infection assays with a clean *mgrB* deletion strain (Δ*mgrB*) supported the hypothesis that this small protein contributes to *Salmonella* virulence not only in macrophages (Fig. 6A), but also in epithelial cells (Fig. S7a). Particularly, deletion of *mgrB* interfered with the ability of *Salmonella* to enter (Fig. 6A, 1 h time point; i.e. before intracellular replication occurs (Steele-Mortimer 2008)) and to replicate inside both host cell types (Fig. 6B, Fig. S7b). The virulence defects of Δ*mgrB* were (over-)complemented by a medium-copy plasmid encoding *mgrB* and its native promoter (*mgrB*^+^ strain, compared to wild-type and Δ*mgrB Salmonella* carrying the empty vector control; Fig. 6A). Of note, the absence of MgrB did not affect *in vitro* growth in either LB or SPI-2 medium (Fig. 6C), arguing for infection-specific effects rather than a general impact on bacterial fitness. Taken together, these data confirm that the small protein MgrB contributes to *Salmonella* infection of two frequently used cell culture models.

### MgrB positively affects the expression of flagella and motility genes

**Figure 7.**
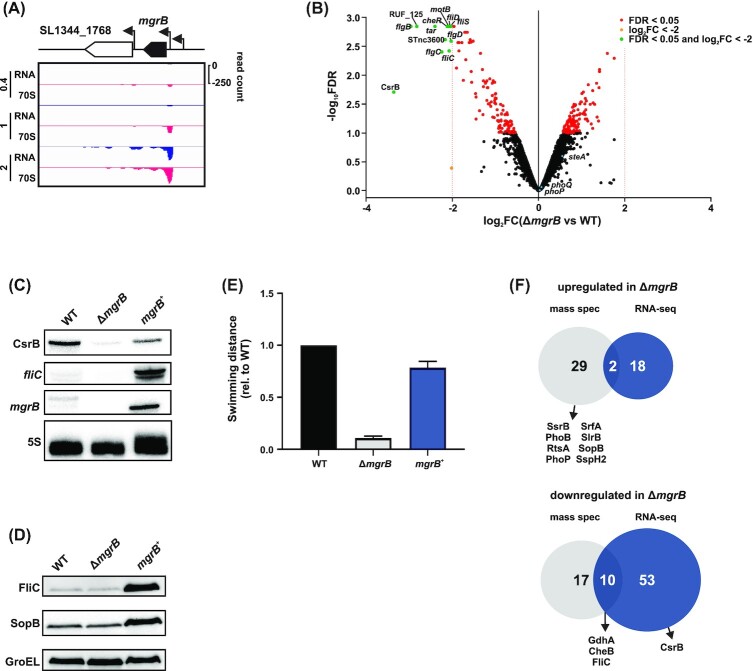
Impact of MgrB on the *Salmonella* transcriptome and proteome. **A**, Expression of *mgrB* as detected by Ribo-seq (RPKM values) in MEP (mid-exponential phase; 0.4), SPI-1- (1) and SPI-2- (2) inducing conditions. RNA = total RNA; 70S = ribosome footprints. **B**, Comparison of the transcriptomes of *Salmonella* wild-type vs Δ*mgrB* grown in SPI-2 medium based on RNA-seq. The transcripts with a significant (FDR < 0.05) dysregulation are coloured in red, while those with a |log_2_FC| > 2 are coloured in orange. RNAs that are dysregulated above the threshold of |log_2_FC| > 2 and significant are highlighted in green. Data are from two biological replicates. **C**, Validation via northern blot of selected transcripts identified as dysregulated by RNA-seq. For this, *Salmonella* wild-type, Δ*mgrB* and *mgrB^+^* were grown in SPI-2-inducing conditions. The figure is representative of three biological replicates. The 5S rRNA was probed for as a loading control. **D**, Validation of the dysregulated proteins FliC and SopB as detected by mass spectrometry in *Salmonella* grown in SPI-2 conditions via western blot. GroEL was probed as a loading control. Replicates are as for panel **C**. **E**, Quantification of swimming distance from the inoculation point of Δ*mgrB* and *mgrB^+^* relative to wild-type in SPI-2 agar. Data were generated from three biological replicates and the error bars represent standard deviations from the mean. **F**, Venn diagrams displaying the overlap between upregulated (top) and downregulated (bottom) proteins or transcripts generated from mass spectrometry and RNA-seq analysis in the presence or absence of *mgrB*. Throughout panels **C** to **F**, wild-type and Δ*mgrB Salmonella* carry the empty vector control.

To identify the molecular features that underlie the effect of MgrB on *Salmonella* virulence, we compared the transcriptomes of *Salmonella* wild-type and the Δ*mgrB* strain grown in SPI-2-inducing medium, a condition where MgrB is highly expressed and translated (Fig. 7A). Our RNA-seq analysis showed that a subset of genes was downregulated in the Δ*mgrB* mutant relative to the wild-type strain (12 genes with log_2_FC < −2, FDR < 0.05, Table S3). These included genes encoding motility- and chemotaxis-related proteins, such as *fliC*, *flgB*, *motB*, *cheR* and *tar* (Fig. 7B).

We independently validated the downregulation of *fliC* mRNA in Δ*mgrB* compared to wild-type *Salmonella* by northern blot analysis (Fig. 7C). Upon overexpression of *mgrB*, we observed an increase in *fliC* mRNA and FliC protein levels on northern and western blots, respectively (Fig. 7C, D). Reduced expression of motility genes in the absence of MgrB lead us to hypothesize that the Δ*mgrB* mutant might have a motility defect. Indeed, the Δ*mgrB* mutant was defective in swimming in SPI-2 medium/0.3% agar plates, an effect that could be complemented in *trans* (Fig. 7E, Fig. S8). This is likely the result of flagellar dysregulation by overactive PhoPQ (which inhibits the flagellar regulon in *Salmonella*; (Fàbrega and Vila 2013)) in the absence of MgrB.

In the comparative transcriptomics data (Fig. 7B), we also noted a strong downregulation of the CsrB sRNA in Δ*mgrB* bacteria. In Enterobacteriaceae, CsrB titrates the global mRNA-binding protein and translational regulator CsrA through its 21 high-affinity CsrA binding sites (Vakulskas *et al*. 2015). We validated the downregulation of CsrB in Δ*mgrB* compared to wild-type *Salmonella* (log_2_FC = −3.37) by northern blot (Fig. 7C). Rifampicin time course experiments suggested that lower CsrB levels in Δ*mgrB Salmonella* are likely the result of reduced sRNA stability in the absence of MgrB (Fig. S9).

### Proteins affected by MgrB deficiency include TCSs and effector proteins

Next, we integrated the differential transcriptomics with proteomics data from the wild-type, Δ*mgrB* and *mgrB*^+^*Salmonella* strains grown under the SPI-2-inducing condition (Table S3; the overlap of the transcriptomic and proteomic datasets is shown in Fig. 7F). Given that *mgrB* levels are higher in the complementation strain than in wild-type *Salmonella* (Fig. 7C) and to prioritize the identification of MgrB-specific effects, we focused on the proteins whose levels were not only significantly altered (*P*-value < 0.01 based on all possible permutations) between Δ*mgrB* vs wild-type, but that also showed opposite dysregulation in *mgrB*^+^ vs wild-type (43 proteins in total). Additionally, we included 15 proteins that were significantly dysregulated in Δ*mgrB* vs wild-type, but showed similar levels in *mgrB*^+^ vs wild-type, in other words with a complementation to wild-type levels rather than an over complementation as for the previous class. Overall, the absence of MgrB lead to a total of 58 dysregulated proteins.

Compared to the wild-type strain, 31 proteins accumulated to higher levels in Δ*mgrB*, including the four T3SS effectors SlrP, SspH2, SrfA and SopB, as well as components of four TCSs (SsrB, PhoB, RstA and PhoP). Despite higher levels of PhoP protein, we did not observe increased *phoP* transcript levels in Δ*mgrB* in the above RNA-seq data (Fig. 7B, Table S3). Instead, higher PhoP protein levels in the *mgrB* mutant strain compared to the wild-type strain might be due to an increased translation rate of *phoP* mRNA or increased protein stability in the absence of this small protein. Conversely, 27 proteins were depleted in Δ*mgrB* compared to both wild-type and *mgrB*^+^*Salmonella*, but enriched (18) or unchanged (9) in the *mgrB*^+^ strain compared to the wild-type. Among them were the motility proteins FliC, CheB and GdhA, which were also repressed at the mRNA level (Fig. 7B). Based on western blot analysis, downregulation of both FliC and SopB in the Δ*mgrB* strain is mild but their levels are over-stabilized in the complementation strain (Fig. 7D).

Finally, in an attempt to disentangle the above molecular changes in Δ*mgrB Salmonella* from PhoP-dependent effects of MgrB, we consulted the SalComRegulon database (Colgan *et al*. 2016). This resource contains RNA-seq data from several *Salmonella* mutants, each devoid of a single global transcriptional regulator or TCS, including a *phoP*-deficient mutant. Comparing the set of dysregulated genes in the transcriptomes of Δ*mgrB* and Δ*phoP Salmonella* revealed large overlaps, but in opposite directions. In the majority of cases, downregulation in Δ*mgrB* corresponded to an upregulation in Δ*phoP* and *vice versa* (examples are shown in Fig. S10). In contrast, other TCSs (e.g. SsrB, PhoB and RstA) were affected at the protein level in the absence of MgrB (Fig. 7F), but their expression was unaffected in Δ*mgrB Salmonella*, which might be an indication for PhoPQ-independent effects of MgrB. Further efforts will be needed to identify alternative direct interaction partners of MgrB besides PhoQ.

## DISCUSSION

It is difficult to overstate the impact of post-genomic technologies on microbiology. It is now often as easy to measure a given functional parameter across the whole genome as it is to assay a single locus. The steady accumulation of these genome-wide datasets presents an opportunity for explorative analyses that integrate this information to produce testable hypotheses. We have taken this approach to small protein discovery and characterization in the model pathogen *Salmonella* Typhimurium, combining new purpose-generated datasets with those from previous studies to identify promising leads for further characterization.

### Refinement of *Salmonella* sORF annotation

To enrich the annotation of the *Salmonella* small proteome, we combined genome-based predictions by sPepFinder (Li and Chao 2020) with ribosome profiling-based data from *Salmonella* grown in well-established conditions mimicking specific stages of infection (host cell invasion and intracellular replication). In this way, we identified 139 novel small proteins, 113 from sPepFinder and 42 from Ribo-seq, that are currently not present in the UniProt or *Salmonella* SL1344 (Kröger *et al*. 2013) annotations. We independently validated translation of 15 out of the 16 STsORFs called by both approaches, as well as two STsORFs highly induced during infection, by epitope-tagging and detection via western blot. These 17 candidates, together with eight predicted in other *Salmonella* strains, represent ‘high-confidence’ new small proteins (Table S2). Indeed, while sPepFinder and Ribo-seq represent valid starting points to create a list of possible sORFs, they are both prone to producing false-positives. Here, we attempted to minimize false-positives by applying additional stringent filters (e.g. mRNA abundance) and validating several candidates by epitope tagging and western blot as a measure for the robustness of the global predictions. However, any of the newly identified candidates should be validated independently using targeted methods like western blot before further study (Table 1).

**Table 1.** Advantages and disadvantages of the approaches used in this work for sORF detection and prioritization (white background) and to be considered when applied to different species (grey background).

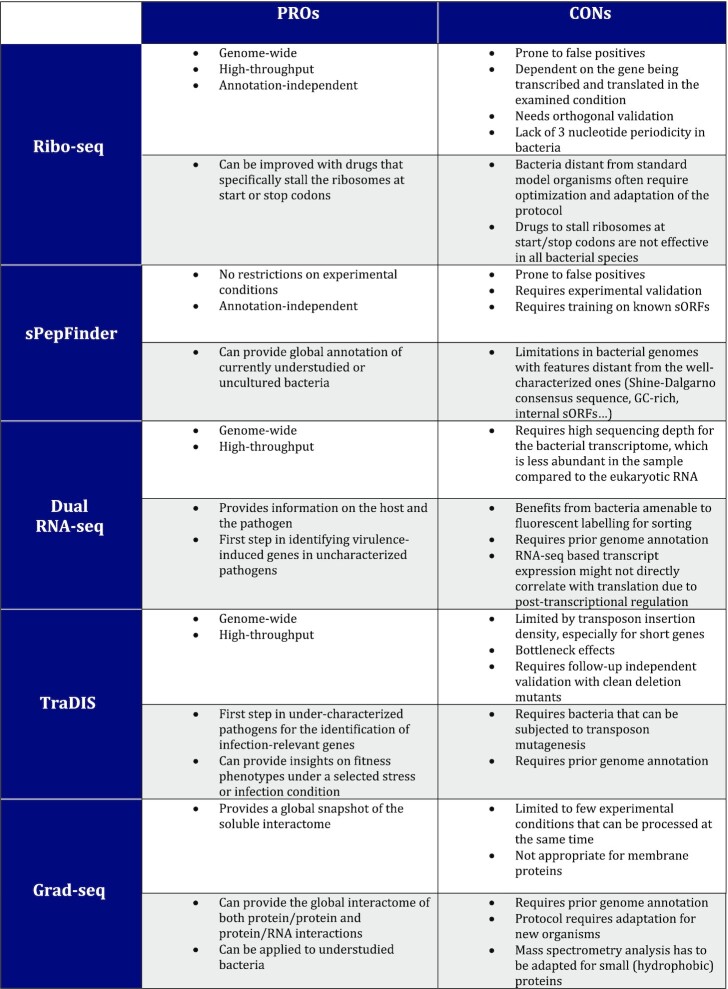

Comparing our results to Ribo-seq-derived sORF predictions in the *Salmonella* Typhimurium strain 14028s (Baek *et al*. 2017) revealed some overlap. For example, seven proteins predicted by sPepFinder, two predicted from our Ribo-seq dataset and three predicted by both approaches were also contained in the updated sORF annotation of strain 14028s. In contrast, 66 sORFs were called exclusively in the 14028s strain, which may be explained by differences in Ribo-seq protocols and data analysis pipelines between the two studies, as well as by genetic differences (Henry, Garcia-Del Portillo and Gorvel 2005; Clark *et al*. 2011). Since *Salmonella* and *E. coli* are closely related, we also examined novel small proteins recently annotated in the *E. coli* MG1655 strain (Weaver *et al*. 2019). Only 11 out of 68 sORFs predicted in *E. coli* resulted in a tBLASTn hit in the *Salmonella* genome with a conservation >50%,  one of which includes a stop codon and one overlaps in-frame with another annotated gene (*yjaB;* data not shown). Among the remaining candidates, only one was called by our approach: STsORF27, predicted by sPepFinder, which is homologous to YljB in *E. coli* (Table S1). This gene was also predicted based on Ribo-seq data in *Salmonella* 14028s (Baek *et al*. 2017). The small overlap between sORFs in *E. coli* and *Salmonella* argues that small protein biology may largely be species-specific or that amino acid conservation barely extends beyond a few highly conserved residues. In summary, after careful analysis of the newly predicted STsORF candidates and cross-comparison with other datasets, we added our 139 predicted STsORFs, including 17 of high-confidence, to the small proteome annotation of *Salmonella* (Table S1).

### Strengths and limitations of several approaches to small protein discovery

We utilized pre-existing or newly generated global datasets to extract functional information on the bulk of *Salmonella* small proteins. Naturally, each of the underlying approaches has its own strengths and limitations when it comes to small proteins (for a summary, see Table 1). For instance, sedimentation profiles of proteins can inform on their potential inter-molecular interactions in the bacterial cytosol. Grad-seq, which relies on mass spectrometry, detected 89 uncharacterized small proteins, although none of our new STsORFs. This reflects the known limitations of mass spectrometry in detecting small proteins in a complex sample. Indeed, without specifically enriching for small proteins, the relatively few peptides that arise after tryptic digestion are diluted out by the peptides derived from average-sized proteins. Therefore, future modifications of the mass spectrometry protocol incorporated into the Grad-seq pipeline, e.g. bypassing the fragmentation step, might increase the chances of detecting small proteins (Gerovac *et al*. 2020). Alternatively, adjusting cut-offs applied for the analysis, such as the requirement for detection of at least two peptides per protein that was applied in this case, might increase the detection of small proteins but will also result in a higher occurrence of false positives (Table 1). Once obtained, small protein sedimentation profiles can provide a fast readout to direct focus on the ones possibly engaged in intermolecular interactions, as a shift to any fraction other than the top two suggests.

We combined dual RNA-seq and TraDIS data to pinpoint potentially virulence-associated small protein candidates of *Salmonella*. For example, *yjiS* was both strongly induced inside epithelial cells and required for bacterial fitness during macrophage infection. However, dual RNA-seq profiles gene expression only on the mRNA level, which does not necessarily translate into changes of the corresponding protein. Further, we note that a TraDIS approach to small proteins is highly dependent on a high transposon insertion density, so targeted approaches like CRISPRi may be preferable in the future (Rousset *et al*. 2018; Wang *et al*. 2018). In addition, follow-up evaluation of the TraDIS data with clean deletion mutants is crucial, e.g. to disentangle the effects of sORFs overlapping with other genomic features from that of adjacent genes.

### The small protein MgrB contributes to *Salmonella* virulence

MgrB was one of the small proteins with a previously investigated molecular function in *Salmonella*. MgrB regulates the activity of the PhoPQ TCS (Lippa and Goulian 2009), a function conserved in *E. coli* (Salazar, Podgornaia and Laub 2016). Indeed, a large fraction of the genes we found differentially expressed in Δ*mgrB Salmonella* were previously shown to be dysregulated in a Δ*phoP* mutant (Colgan *et al*. 2016).

Here, we showed for the first time that, relative to the isogenic wild-type strain, Δ*mgrB Salmonella* is impaired at all stages of macrophages and epithelial cells infection. We uncovered a positive effect of MgrB on flagella and motility-related genes at both the transcript and protein level. Upon further inspection of the data, we linked this effect to PhoPQ, well known for its ability to repress flagella synthesis (Adams *et al*. 2001), likely reinforced through the PhoP target operon *ssrAB*. In our proteomics data the histidine kinase SsrB was upregulated in the Δ*mgrB* mutant. Besides its activity as the master regulator of the SPI-2 regulon (Walthers *et al*. 2007), SsrB interferes with transcription of *flhDC*, encoding the master regulators of the flagellar expression cascade by activating the transcription regulator SsrA (Ilyas *et al*. 2018). This could therefore contribute to the observed inhibition of flagellar gene expression in the absence of MgrB. Furthermore, elevated SsrB levels are known to lead to a defect in epithelial cell invasion (Pérez-Morales *et al*. 2017), another phenotype we found associated with the lack of MgrB.

MgrB also affected the expression of certain genes that are currently not considered direct members of the PhoPQ regulon. This suggests additional, PhoPQ-independent roles for MgrB, supported by recent findings of *E. coli* MgrB interacting with additional histidine kinases such as PhoR (Yadavalli *et al*. 2020). In our proteomics data, we observed PhoB, the response regulator of the PhoBR TCS, to be de-repressed in the Δ*mgrB* mutant. As histidine kinases and response regulators of different TCSs share several conserved domains, crosstalk between such systems has been hypothesized (Laub and Goulian 2007). It is therefore tempting to speculate that MgrB could act as a regulator of different histidine kinases and thus mediate the cross-talk between the respective target regulons. Uncoupling PhoPQ-dependent from PhoPQ-independent effects will be necessary to further asses the role(s) of MgrB in *Salmonella* virulence, but may be aggravated by the fact that *mgrB* is itself a member of the PhoPQ regulon.

## CONCLUSIONS

Our integrative approach to identifying and prioritizing small proteins for further study in *Salmonella* will serve as a blueprint for other species. We have compiled an overview of the individual strengths and limitations of these genome-wide approaches in the context of bacterial small protein research, and mention important aspects to consider when generating *de novo* data for purposes that involve small proteins (Table 1). Countless global datasets are available for diverse bacterial organisms including Gram-positive species. For example, high-resolution transcriptomics, transposon mutagenesis and Grad-seq data exist for *Streptococcus pneumoniae* (van Opijnen and Camilli 2012; Aprianto *et al*. 2016, 2018; Warrier *et al*. 2018; Rowe *et al*. 2019; Hör *et al*. 2020b). More generally, various transposon-sequencing approaches have been applied to bacterial pathogens under virulence conditions (Karlinsey *et al*. 2019; Warr *et al*. 2019; Cain *et al*. 2020; Rendón *et al*. 2020) and dual RNA-seq has become the gold-standard to chart the transcriptional landscape of pathogens during infection (Westermann, Barquist and Vogel 2017; Montoya *et al*. 2019; Ritchie and Evans 2019; Pisu *et al*. 2020). Re-inspection of these data will provide invaluable information on potentially new biological roles carried out by small proteins in bacterial pathogenesis.

## METHODS

### Strains and growth conditions

All bacterial strains and plasmids used for this study are reported in Table S4. The Δ*mgrB* strain, as well as the SPA-tagged strains, were generated as previously described (Datsenko and Wanner 2000). Oligonucleotides used for cloning can be found in Table S4. The respective mutations were then transduced in the wild-type or green fluorescent protein(GFP^+^) background using P22 phages (Sternberg and Maurer 1991). For routine growth of *Salmonella*, a single bacterial colony was grown overnight in LB medium at 37°C with shaking at 220 rpm, diluted 1:100 into fresh medium and then grown to the required cell density. The SPI-1-inducing condition is defined as growth in LB to an OD_600_ of 2.0 (Kröger *et al*. 2013). For growth in SPI-2-inducing conditions (Löber *et al*. 2006), cells that had reached SPI-1 conditions were centrifuged for 2 min at 12,000 rpm at room temperature (RT), washed twice with PBS (Gibco) and once with SPI-2 medium (Löber *et al*. 2006), and then diluted 1:50 into fresh SPI-2 medium. The cultures were again grown at 37°C and 220 rpm to an OD_600_ 0.3. When required, the medium was supplemented with 100 μg/ml ampicillin.

### Mammalian cell cultures

HeLa-S3 cells (ATCC CCL-2.2) and RAW264.7 mouse macrophages (ATCC TIB-71) were cultured as described in (Westermann *et al*. 2016). HeLa cells were passaged in DMEM (Gibco) and RAW264.7 cells were passaged in RPMI (Gibco) medium supplemented with 10% fetal calf serum (FCS, Biochrom), 2 mM L-glutamine (Gibco) and 1 mM sodium pyruvate (Gibco) in T-75 flasks (Corning). Cells were grown in a 5% CO_2_, humidified atmosphere at 37°C and routinely tested for mycoplasma contamination with the MycoAlert Mycoplasma Detection kit (Lonza). Two days before infection, 2 × 10^5^ cells were seeded in six-wells plates (2 ml medium).

### sPepFinder

sPepFinder is a SVM learning-based computational approach for *ab initio* prediction of bacterial sORFs (Li and Chao 2020). Briefly, it first extracts informative features from a collection of sequence and structural properties of known bacterial small proteins. The informative features include a thermodynamic model of ribosome binding sites and amino acid composition. sPepFinder has achieved a 92.8% accuracy in 10-fold cross validation in a test dataset of ten bacterial species (eight from the Enterobacteriaceae family, as well as *Vibrio cholerae* and *Pseudomonas*).

### Ribosome profiling

Ribosome profiling was performed as previously described (Oh *et al*. 2011) with modifications. *Salmonella* wild-type was grown in LB, SPI-1- and SPI-2-inducing conditions as described above. Bacterial cells were harvested by fast-filtration with a 0.45 µm polyethersulfone membrane (Millipore) and immediately frozen in liquid N_2_. Before harvesting, a sample was taken for total RNA analysis, mixed with 0.2 vol stop mix (5% buffer-saturated phenol (Roth) in 95% ethanol). Cell pellets were resuspended in ice-cold lysis buffer (100 mM NH_4_Cl, 10 mM MgCl_2_, 20 mM Tris-HCl, pH 8.0, 0.1% NP-40, 0.4% Triton X-100 (Gibco), 50 U/ml DNase I (Fermentas), 500 U RNase Inhibitor (moloX, Berlin), 1 mM chloramphenicol) and lysed using glass beads and vortexing at high speed for 10 × 30 s, with chilling on ice in between each round. Lysates were clarified by centrifugation at 21 000*g* for 10 min. Next, 14–17 A_260_ of lysate was digested with 800 U/A_260_ of micrococcal nuclease (MNase, NEB) at 25°C with shaking at 14 500 rpm for 20 min in lysis buffer supplemented with 2 mM CaCl_2_ and 500 U RNase Inhibitor. A mock-digested control (no enzyme added) was also included for each lysate to confirm the presence of polysomes. Digests were stopped with ethylene glycol-bis(β-aminoethyl ether)-*N*, *N*, *N*′, *N*′-tetraacetic acid (EGTA, final concentration 6 mM) and immediately loaded onto 10%–55% sucrose gradients prepared in sucrose buffer (100 mM NH_4_Cl, 10 mM MgCl_2_, 5 mM CaCl_2_, 20 mM Tris-HCl, pH 8.0, 1 mM chloramphenicol) with 2 mM fresh dithiothreitol. Gradients were centrifuged in a SW40-Ti rotor in a Beckman Coulter Optima L-80 XP ultracentrifuge for 2 h 30 min at 35 000 rpm at 4°C, and then 70S monosome fractions were collected using a Gradient Station *ip* (Biocomp Instruments). RNA was extracted from fractions or cell pellets for total RNA using hot phenol-chloroform-isoamyl alcohol (25:24:1, Roth) or hot phenol, respectively, as described previously (Sharma *et al*. 2007; Vasquez *et al*. 2014). Ribosomal RNA was depleted from 5 µg of total RNA by subtractive hybridization with a complex probe set for *Salmonella enterica* (Senterica_riboPOOL-RP1, siTOOLs, Germany) according to the manufacturer's protocol with Dynabeads MyOne Streptavidin T1 beads (Invitrogen). Total RNA was fragmented with RNA Fragmentation Reagent (Ambion). Monosome RNA and fragmented total RNA was size-selected (26–34 nt) on 15% polyacrylamide/7 M urea gels as described previously (Ingolia *et al*. 2012) using RNA oligonucleotides NI-19 and NI-20 as guides. RNA was cleaned up and concentrated by isopropanol precipitation with 15 μg GlycoBlue (Ambion) and dissolved in H_2_O. Libraries were prepared by Vertis Biotechnologie AG (Freising, Germany) using a small RNA protocol without fragmentation and sequenced on a NextSeq500 instrument (high-output, 75 cycles) at the Core Unit SysMed at the University of Würzburg.

### Analysis of ribosome profiling data

Read files were processed and analyzed with HRIBO (version 1.4.3) (Gelhausen *et al*. 2020), a snakemake (Köster and Rahmann 2012) based workflow that downloads all required tools from bioconda (Grüning *et al*. 2018) and automatically determines the necessary processing steps. We additionally used pinned tool versions which ensures reproducibility of the analysis. Adapters were trimmed from the reads with cutadapt (version 2.1) (Martin 2011) and then mapped with segemehl (version 0.3.4) (Otto, Stadler and Hoffmann 2014). Reads mapping to ribosomal RNA genes and multi-mappers were removed with SAMtools (version 1.9) (Li *et al*. 2009) using the rRNA annotation. Open reading frames were called with an adapted variant of REPARATION (Ndah *et al*. 2017) which uses blast instead of usearch (see https://github.com/RickGelhausen/REPARATION_blast). Quality control was performed by creating read count statistics for each processing step and RNA-class with Subread featureCounts(1.6.3) (Liao, Smyth and Shi 2014). All processing steps were analysed with FastQC (version 0.11.8) (Andrews) and results were aggregated with MultiQC (version 1.7) (Ewels *et al*. 2016). Summary statistics for all available annotated and merged novel ORFs detected by REPARATION were computed in a tabularized form including translational efficiency, reads per kilobase million (RPKM) normalized readcounts, codon counts, nucleotide and amino acid sequences, etc. Additionally, GFF track files with the same information were created for in-depth genome browser inspection, in addition to GFF files showing potential start/stop codon and RBS information. While sORFs predictions were generated based on one replicate, we generated a second replicate to add robustness to our predictions. We then analysed both replicates to filter out sORFs (both new and annotated) that did not match the following criteria: at least 6 RPKM (reads per kilobase million) in the total RNA of both replicates of at least one growth condition, and at least 10 RPKM in one replicate of the ribosome footprints in the corresponding growth condition. This filtering applied to known sORFs showed translation of 356 sORFs out of 470. We filtered predicted STsORFs with the same parameters, and visually inspected sequence coverages for reads accumulation upstream the putative start codon. The only exception to this was STsORF111, included in the Ribo-seq predictions because it ranked highly in the sPepFinder predictions.

### Dual RNA-seq

Data were taken from (Westermann *et al*. 2016). In brief, GFP+ *Salmonella* was used to infect HeLa cells (HeLa-S3; ATCC CCL-2.2) with an multiplicity of infection (MOI) of 5. At different time points (2, 4, 8, 16, 24 h p.i.) cells were collected and sorted to enrich for infected epithelial cells (GFP+). These cells were subjected to RNA extraction and sequencing after rRNA depletion. RNA sequencing was also performed on *Salmonella* grown in LB to OD_600_ 2.0, which represents the inoculum used for infection. Re-analysis of the data was carried out as in (Westermann *et al*. 2016) with our updated annotation.

### TraDIS

A *Salmonella* transposon mutant library containing circa 100 000 mutants was generated using EZ-Tn5 transposase (Epicentre) and the *aphA1* kanamycin resistance gene as described previously (Langridge *et al*. 2009). Two days before infection, RAW264.7 cells were seeded at a density of 2 × 10^6^ in two 75 ml flasks per replicate in RPMI supplemented with penicillin and streptomycin, and then changed to an antibiotic free medium one day before infection. An aliquot of 1 OD_600_/ml equivalent of the *Salmonella* library was grown in 200 ml LB with 10 μg/ml kanamycin at 37°C overnight with shaking. Next, 2 OD_600_ equivalents of this overnight culture were pelleted and resuspended in RPMI with 10% mouse serum for 20 min at RT for opsonization. A similar amount of overnight culture was pelleted and used for input genomic DNA preparation. The RAW264.7 cells were then infected directly in flasks at an MOI of 20, centrifuged for 10 min at 250*g* at RT and incubated for 30 min at 37°C. The medium was then replaced with RPMI containing 100 μg/ml gentamicin, incubated for an additional for 30 min, then washed with PBS and replaced again with RPMI containing 10 μg/ml gentamicin for the remaining duration of the experiment. At 20 h p.i., the medium was aspirated, and cells were washed once with PBS before being scraped from the flask in 10 ml PBS and harvested by centrifugation for 10 min at 250*g*. The supernatant was discarded, and samples for each replicate were pooled in 6 ml PBS containing 0.1% (v/v) Triton X-100. This was incubated for 10 min at RT with occasional vortexing, before being centrifuged again at 250*g* for 10 min. The supernatant was recovered, pelleted, and used for DNA extraction. DNA was extracted using the phenol-chloroform method. Briefly, bacterial pellets were resuspended in 250 μl of a 50 mM Tris-HCl, 50 mM EDTA, pH 8 solution and frozen for at least 1 h at −20°C. Pellets were then defrosted at RT and treated with 2.5 μg/ml lysozyme on ice for 45 min, followed by 2.4 μg/ml per OD unit of input culture RNase A (Fermentas) for 40 min at 37°C, and then ∼333 μg/ml proteinase K in buffer (0.5% (w/v) SDS, 50 mM Tris-HCl, 0.4 M EDTA, pH 8) at 50°C until the sample cleared (approx. 30 min–1h). This solution was then mixed with 300 μl milliQ filtered water and added to a phase lock gel (PLG) tube containing 400 μl phenol/chloroform (Roth). The sample was vigorously mixed by inversion, then centrifuged at 15°C for 15 min. The aqueous phase was collected, then precipitated with 1.4 ml 100% ethanol containing 0.1 M sodium acetate and inverted 6–8 times. This solution was then centrifuged at 13,000 rpm at RT for 20 min, the supernatant was discarded, and the pellet was washed with 70% ethanol before drying and resuspension in milliQ filtered water. Sequencing of 2 replicate infection experiments was performed at the Wellcome Trust Sanger Institute using the TraDIS dark-cycle sequencing protocol for 50 cycles on a MiSeq sequencer (Illumina) with a read count yield of between 1.3 and 1.9 million reads as previously described (Barquist *et al*. 2016). The reads were then processed with the Bio-TraDIS Toolkit (https://github.com/sanger-pathogens/Bio-Tradis, (Barquist *et al*. 2016)), with ∼98%–99% of reads matching the expected transposon tag sequence, and subsequent read mapping rates of ∼94%–98%. Transposon read counts per gene were then summarized using the tradis_gene_insert_sites script, excluding insertions in the first and last 10% of each gene. Infected samples were then compared to controls using the tradis_comparison.R script, using edgeR (Robinson, McCarthy and Smyth 2010), and filtered for genes with a |log_2_FC| > 1 and a q-value < 0.05.

### Grad-seq

Gradient profiling mass spectrometry data were taken from (Gerovac *et al*. 2020), based on the Grad-seq approach first described in (Smirnov *et al*. 2016). In brief, *Salmonella* wild-type was grown in LB to an OD_600_ of 2.0, collected, and lysed by glass bead beating. The lysate was then loaded on a linear glycerol gradient (10%–40% w/v) and separated by ultracentrifugation. The gradient was then fractionated and each fraction, including the pellet, was analysed by mass spectrometry.

### RNA extraction

For total RNA preparation, cells were grown in SPI-2 medium to an OD_600_ of 0.3 as previously described. Then, 10 ml of culture were mixed with 2 ml of STOP solution (95% ethanol, 5% phenol), snap frozen in liquid nitrogen and total RNA was extracted via the ‘hot-phenol’ method (Vasquez *et al*. 2014). Briefly, the frozen culture was thawed, centrifuged 20 min at 4500 rpm and 4°C and the cell pellet resuspended in 0.5 mg/ml lysozyme in TE, pH 8.0. Next, 60 μl of 10% w/v SDS was added, the tube was mixed by inversion, and incubated for 2 min at 64°C. After this, 66 μl of 3 M sodium acetate, pH 5.2 was added, followed by 750 μl phenol (Roti Aqua phenol, Roth). The solutions were mixed by inversion and incubated 6 min at 64°C. Tubes were then cooled on ice and centrifuged 15 min at 13,000 rpm at 4°C. The aqueous phase was then moved to 2 ml PLG tubes, to which 750 μl chloroform were added, shaken and centrifuged 12 min 13,000 rpm at 4°C. The aqueous phase was moved to new tubes to which were added 2 volumes of 30:1 mix (ethanol:3 M sodium acetate, pH 6.5). This was left to precipitate over night at −20°C, then centrifuged for 30 min at 4°C and 13,000 rpm, washed with 75% v/v ethanol, and resuspended in RNase-free water by shaking for 5 min at 65°C. To remove DNA contamination, samples were treated with 0.25 U of DNase I (Fermentas) for 1 μg RNA for 45 min at 37°C.

### Total RNA-seq and analysis

Complementary DNA (cDNA) libraries of total RNA were prepared at Vertis Biotechnologie AG after rRNA depletion (Ribo-Zero rRNA Removal Kit (Bacteria); Illumina). Sequencing was performed on an Illumina NextSeq 500 platform with approximately 20 million reads per library. The adapters were removed from the FASTQ format reads using cutadapt, then quality trimming was carried out with fastq_quality_trimmer from the FastX suite (Version 0.3.7). Alignment to the *Salmonella* Typhimurium SL1344 genome downloaded from NCBI (NC_01 6810, NC_01 7718, NC_01 7719 and NC_01 7720) was performed using READemption (Förstner, Vogel and Sharma 2014) (Version 0.4.5). Differential gene expression analysis was carried out with edgeR (Robinson, McCarthy and Smyth 2010) (Version 3.20.8). Only genes with at least 5 uniquely mapped reads in two experiments were considered. The cut-off for differential expression was set to q-value < 0.05 and |log_2_FC| > 2.

### Northern blot analysis

Northern blot analysis of 10 μg of RNA per sample was performed with 6% (v/v) polyacrylamide-7 M urea gels as previously described (Westermann *et al*. 2016). ^32^P-labelled DNA oligonucleotides (Table S4) complementary to the transcript of interest was then incubated in Hybri-Quick buffer (Carl Roth AG) on the membranes (Hybond XL membranes, Amersham) at 42°C overnight, followed by sequential washes in SSC buffers (5x, 1x, 0.5x) with 0.1% SDS. The membranes were then exposed on phosphor screen and revealed on a Typhoon FLA 7000 phosphorimager (GE Healthcare). Signal quantification was performed using ImageJ (Schneider, Rasband and Eliceiri 2012).

### Western blotting

For western blot validation of novel STsORFs and mass spectrometry data, cells were grown to the appropriate OD_600_ in SPI-1 or SPI-2 medium, harvested by centrifugation at RT for 2 min at 16,000*g*,  and resuspended in 1x protein loading buffer (5x solution: 5g SDS, 46.95 ml Tris‐HCl, pH 6.8, 0.075 g bromophenol blue, 75 ml glycerol, 11.56 g dithiothreitol, filled up to 150 ml H_2_O) at a concentration of 0.01 OD/μl. Samples were denatured for 5 min at 95°C and 0.1 OD equivalents were separated on 15% SDS-PAGE gels. Separated proteins were then transferred to a PVDF membrane (Perkin Elmer) for 90 min with a semidry blotter (Peqlab; 0.2 mA/cm^2^ at 4°C) in transfer buffer (25 mM Tris base, 190 mM glycine, 20% (v/v) methanol). After transfer, membranes were blocked for 1 h at RT in 10% (w/v) milk/TBS-T20 (Tris-buffered-saline-Tween-20). The membranes were then incubated with the appropriate primary antibody (Table S4) at 4°C overnight, and then after three 5 min washes with TBS-T20, incubated with the corresponding secondary antibody (Table S4) for 1 h at RT. At the end of the hybridization, the membranes were again washed three times for 5 min with TBS-T20 and the blots developed with western lightning solution (Perkin Elmer) with a Fuji LAS-4000 imager (GE Healthcare).

### Whole proteome preparation

For the preparation of the total *Salmonella* proteome for mass spectrometry analysis, cells were grown in SPI-2-inducing conditions (see above). At an OD_600_ of 0.3, cells were pelleted, washed, and resuspended in protein loading dye for loading on a precast gel at a concentration of 1 OD/100 μl. Proteins were separated by 1D SDS-PAGE and prepared for MS/MS analyses as previously described (Bonn *et al*. 2014). Briefly, gel lanes were fractionated into 10 gel pieces, cut into smaller blocks and transferred into low-binding tubes. Samples were washed until gel blocks were destained. After drying of gel pieces in a vacuum centrifuge, they were covered with trypsin solution. Digestion took place at 37°C overnight before peptides were eluted in water by ultrasonication. The peptide-containing supernatant was transferred into a fresh tube, desiccated in a vacuum centrifuge and peptides were resolubilized in 0.1% (v/v) acetic acid for mass spectrometry analysis.

### MS/MS analysis

Tryptic peptides were subjected to liquid chromatography (LC) separation and electrospray ionization-based mass spectrometry applying exactly the same injected volumes in order to allow for label-free relative protein quantification. Therefore, peptides were loaded on a self-packed analytical column (OD 360 μm, ID 100 μm, length 20 cm) filled with of Reprosil-Gold 300 C18, 5 µm material (Dr. Maisch, Ammerbuch-Entringen, Germany) and eluted by a binary nonlinear gradient of 5%–99% acetonitrile in 0.1% acetic acid over 83 min with a flow rate of 300 nl/min. LC-MS/MS analyses were performed on an LTQ Orbitrap Elite (ThermoFisher Scientific, Waltham, Massachusetts, USA) using an EASY-nLC 1200 LC system. For mass spectrometry analysis, a full scan in the Orbitrap with a resolution of 60,000 was followed by collision-induced dissociation of the twenty most abundant precursor ions. MS2 experiments were acquired in the linear ion trap.

### MS data analysis

A database search against a *Salmonella* Typhimurium SL1344 annotation downloaded from Uniprot (date 23/08/2018, organism ID 216597, 4659 entries) as well as label-free quantification (LFQ) was performed using MaxQuant (version 1.6.2.6) (Cox and Mann 2008). Common laboratory contaminants and reversed sequences were included by MaxQuant. Search parameters were set as follows: Trypsin/P specific digestion with up to two missed cleavages, methionine oxidation (+15.99 Da) as a variable and carbamidomethylation at cysteines (+57.02 Da) as fixed modification, match between runs with default parameters enabled. The FDRs of protein and peptide spectrum match levels were set to 0.01. Two identified peptides with at least one of those being unique were required for protein identification. LFQ was performed using the following settings: LFQ minimum ratio count 2 considering only unique peptides for quantification. Results were filtered for proteins quantified in at least two out of three biological replicates before statistical analysis. Here, two strains (of either wild-type, Δ*mgrB* and *mgrB*+) were compared by a student's *t*-test applying a threshold *P*-value of 0.01, which was based on all possible permutations.

### Infection assay, colony forming unit counting and flow cytometry

For infection of HeLa cells, overnight cultures were diluted 1:100 in fresh LB medium (supplemented with the appropriate antibiotic if needed) and grown aerobically to an OD_600_ of 2.0. Bacteria were collected by centrifugation (2 min at 12,000 rpm, RT) and resuspended in DMEM. HeLa cells were infected at a MOI of 25. After addition of the bacteria, cells were centrifuged at 250*g* for 10 min at RT and subsequently incubated at 37°C in 5% CO_2_ and humidified atmosphere. After this, the medium was exchanged (this step marking the time zero) with one containing 50 μg/ml gentamicin for 30 min to kill extracellular bacteria, and then changed again to 10 μg/ml for the remainder of the infection. Cells were collected at the indicated time points. Colony forming unit (CFU) assays were performed to quantify intracellular bacteria. At the indicated time points, the infected cells were washed twice with PBS and lysed in PBS with 0.1% (v/v) Triton X-100. The lysates were diluted in PBS and plated on LB agar plates (with appropriate antibiotic if required) and incubated overnight at 37°C. CFUs were then counted and normalized to the number of the bacterial inoculum used for the infection and fold-changes relative to the wild-type strain for each time point were calculated. The flow cytometry assay was used to quantify the bacterial intracellular replication. GFP-expressing *Salmonella* were used for infection of HeLa cells as described above. Infected cells were detached at the indicated time points by trypsin digestion (manufacturer), washed with cold PBS by centrifugation (5 min, 550*g* at 4°C) and analysed with a FACS BD Accuri^TM^ C6 (BD Biosciences) instrument, collecting at least 20,000 GFP-positive cells for each condition. Fluorescence was then compared to 1 h-infected cells. The data were analysed with the FlowJo software (Tree Star Inc.). Infection of RAW264.7 macrophages was carried out as in the TraDIS protocol.

### Motility assay

For swimming assays, 6 μl of overnight cultures in LB or SPI-2 minimal medium were spotted at the centre of 0.3% agar SPI-2 plates, in the presence of the appropriate antibiotic if required. Swimming was monitored after 6 h of incubation at 37°C.

### Rifampicin assay

For the RNA stability assay, rifampicin was added at a final concentration of 500 μg/ml to liquid cultures in SPI-2 medium at an OD_600_ 0.3. Samples for total RNA were collected at the indicated time points following rifampicin addition, and then RNA was extracted and analysed by northern blot as described above.

## DECLARATIONS

### Ethics approval

Not applicable.

### Consent for publication

Not applicable.

### Availability of data and materials

sPepFinder predictions analysed here were taken from (Li and Chao 2020): sPepFinder is available at https://github.com/LeiLiSysBio/sPepfinder.

Ribo-seq data that support the findings of this study have been deposited in GEO under the accession number GSE149893.

TraDIS data that support the findings of this study have been deposited in GEO under the accession number GSE149580.

RNA-seq data that support the findings of this study have been deposited in GEO under the accession number GSE149928.

Mass spectrometry data generated in this work that support the findings of this study have been deposited in PRIDE (Perez-Riverol *et al*. 2019) under the accession number PXD018754.

Mass spectrometry data from GradR analysed in this work that support the findings of this study have been deposited in PRIDE (Perez-Riverol *et al*. 2019) under the accession number PXD018422.

The dual RNA-seq data analysed here were taken from (Westermann *et al*. 2016), DOI:10.1038/nature16547.

## AUTHORS’ CONTRIBUTIONS

EV, AJW and JV designed the research. EV, SLS, AKC performed lab work. EV, SM, RG, FE, LL and LB analysed data. EV, SLS, AJW and JV interpreted the data and wrote the manuscript, which all co-authors revised.

## Supplementary Material

uqaa002_Supplemental_Files
