# Supplementary material for: A global data-driven census of Salmonella small proteins and their potential functions in bacterial virulence
Source: Microlife. 2020 Oct 17;1(1):uqaa002. doi: 10.1093/femsml/uqaa002 (PMC10117436; doi:10.1093/femsml/uqaa002)
Supplement: uqaa002_Supplemental_Files [file uqaa002_supplemental_files.zip › Fig.S2.pdf]

Figure S2

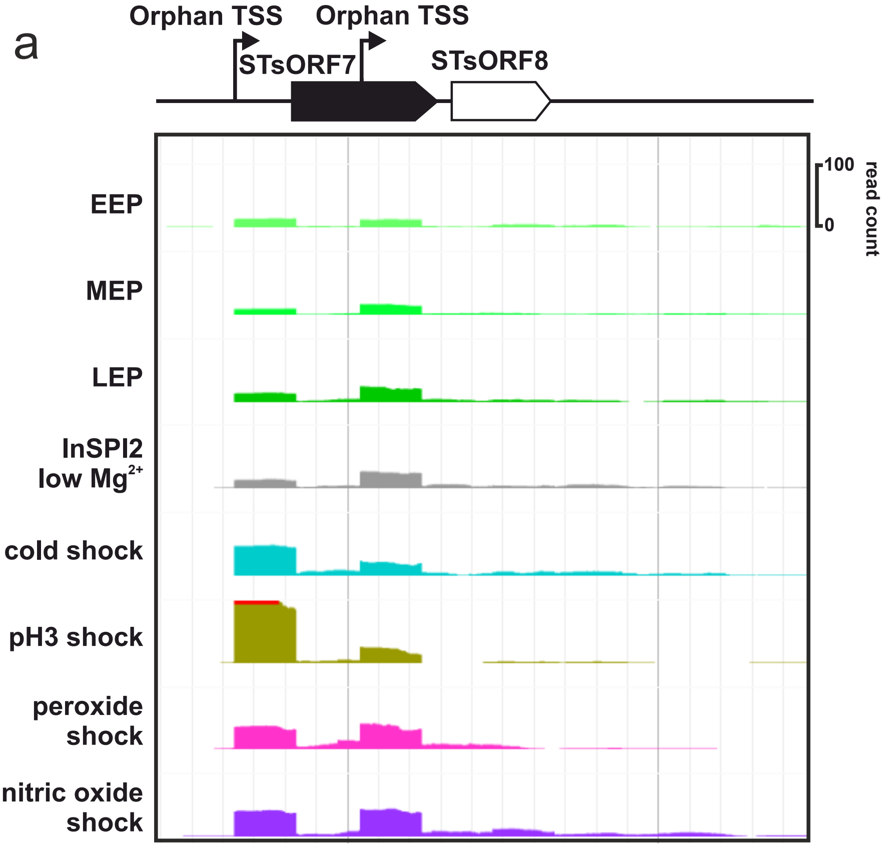

STsORF7

```
       M  K  I  L  N  N  N  T  I  I  V  G  M  E  I  H  L  L  A  K  L  P  H  V  R  F  H  F  Y  V  M
SL  ATGAAGATTTTAAATAACAATACGATAATCGTCGGTATGGAAATCCATCTCCTCGCCAAATTGCCCCACGTACGGTTTCACTTCTACGTTATGTAA
ST  ATGAAGATTTTAAATAACAATACGATAATCGTCGGTATGGAAATCCATCTCCTCGCCAAATTGCCCCACGTACGGTTTCACTTCTACGTTGTGTAA
SE  ATGAAAATTTTAAATAACAATACGATAACCGTCGGTATGGAAATCCATCTCCTCGCCAAATTGCCCCACGTACGGTTTCACTTCTACGTTATGTAA
SG  ATGAAGATTTTAAATAACAATACGATAATCGTCGGTATGGAAATCCATCTCCTCGCCAAATTGCCCCACGTACGGTTTCACTTCTACGTTATGTAA
```

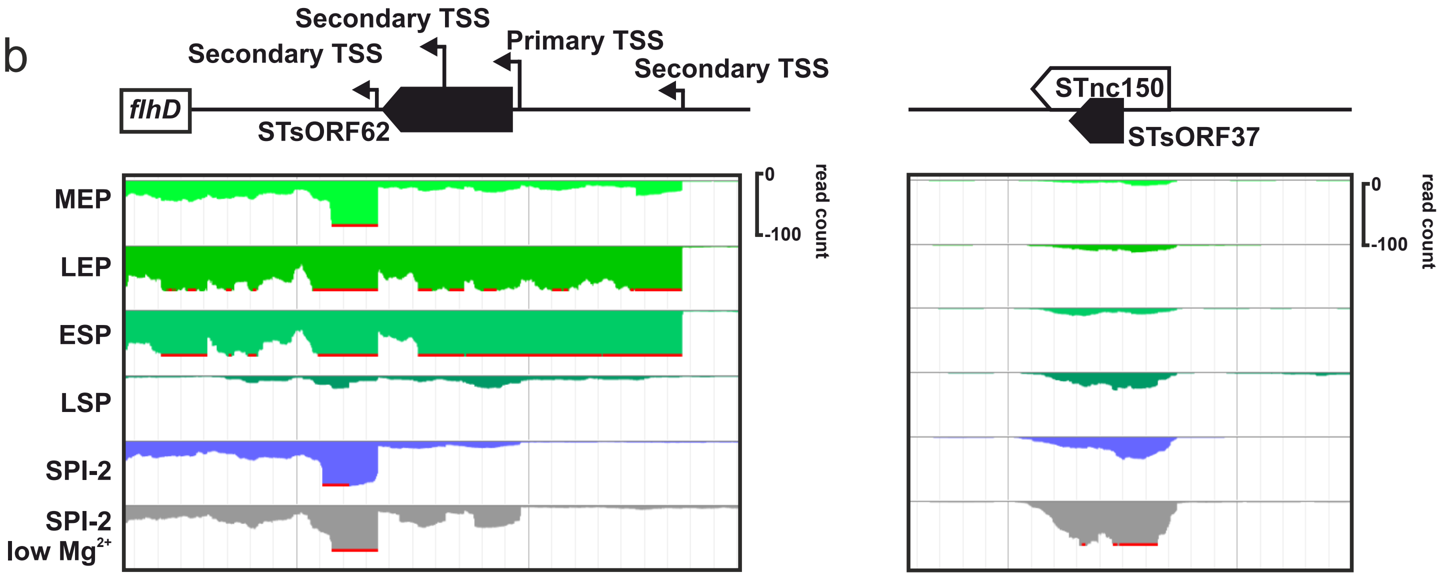

STsORF62

```
       M  N  I  R  L  L  I  K  Q  S  K  S  H  A  D  G  F  I  V  S  I  P  L  K  Y  V  I  C  I  T  Y  F  L  K  S  P  S  R  S  V  V  C  H  E  A  D  E
SL  GTGAACATTAGGTTATTAATTAAACAAAGTAAAAGCCATGCTGATGGTTTTATCGTAAGTATTCCGTTAAAATATGTGATCTGCATCACATATTTTCTAAAATCGCCGTCCCGCTCCGTTGTATGTCACGAAGCTGACGAGTAG
ST  GTGAATATTAGGTTATTAATTAAACAAAGTAAAAGCCATACTGATGGTTTTATCGTAAGTATTCTGTTAAATTATGTGATCTGCATCGCATATTTTCTAAAATCGCCGTCCCGCTCCGTTGTATGTCACGAAACTGACGAGTAG
SE  GTGAACATTAGGTTATTAATTAAACAAAGTAAAAGCCATGCTGATGGTTTTATCGTAAGTATTCCGTTAAAATATGTGATCTGCATCACATATTTTCTAAAATCGCCGTCCCGCTCCGTTGTATGTCACGAAGCTGACGAGTAG
SG  GTGAACATTAGGTTATTAATTAAACAAAGTAAAAGCCATGCTGATGGTTTTATCGTAAGTATTCCGGTAGATTATGTGATCTACATCACATATTTTCTAAAATCGCCGTCCCGCTCCGTTGTATGTCACGAAGCTGACGAGTAG
```

STsORF37

```
       M  T  I  G  V  K  D  E  L  L  L  R  S  E  L  R  E  F
SL  ATGACGATCGGGGTAAAGGATGAACTACTATTGCGGTCTGAATTGAGGGAGTTTTGA
SE  ATGACGATCGGGGTAAAGGATGAACTACTATTGCGGTCTGAATTGAGGGAGTTTTGA
SG  ATGACGATCGGGGTAAAGGATGAACTACTATTGCGGTCTGAATTGAGGGAGTTTTGA
SB  ATGACGATCGGGGTAAAGGATGGATTACTATTGCGGTCTGAATTGAGGGAGTTTTGA
```
