# Supplementary material for: A global data-driven census of Salmonella small proteins and their potential functions in bacterial virulence
Source: Microlife. 2020 Oct 17;1(1):uqaa002. doi: 10.1093/femsml/uqaa002 (PMC10117436; doi:10.1093/femsml/uqaa002)
Supplement: uqaa002_Supplemental_Files [file uqaa002_supplemental_files.zip › Fig.S3.pdf]

Figure S3

STsORF23

```
    M  N  E  F  K  R  C  I  R  V  F  S  H  S  P  F  K  V  R  L  M  L  L  S  M  L  C  D  M  I  N  G  K  P  E  Q  D  N  P  S  T  K
SL  ATGAACGAATTTAAGAGGTGTATACGCGTGTTCAGTCACTCTCCCTTTAAAGTCCGGTTAATGCTGCTCTCTATGTTGTGCGATATGATCAACGGCAAACCGGAGCAGGATAACCCTTCCACGAAATAA
ST  ATGAACGAATTTAAGAGGTGTATACGCGTGTTCAGTCACTCTCCCTTTAAAGTCCGGTTAATGCTGCTCTCTATGTTGTGCGATATGATCAACGGCAAACCGGAACAGGATAACCCTTCCACGAAATAA
SE  ATGAACGAATTTAAGAGGTGTATACGCGTGTTCAGTCACTCTCCCTTTAAAGTCCGGTTAATGCTGCTCTCTATGTTGTGCGATATGATCAACGGCAAACCGGAGCAGGATAACCCTTCCACGAAATAA
SG  ATGAACGAATTTAAGAGGTGTATACGCGTGTTCAGTCACTCTCCCTTTAAAGTCCGGTTAATGCTGCTCTCTATGTTGTGCGATATGCTCAACGGCAAACCGGAGCAGGATAACCCTTCCACGAAATAA
EC  ATGAATGAGTTCAAGAGGTGTATGCGCGTGTTTAGTCATTCTCCCTTTAAAGTACGGTTAATGCTGCTCTCTATGTTGTGCGATATGGTCAACAACAAACCGCAGCAAGATAAACCTTCCGATAAATAA
```

STsORF44

```
    M  K  R  F  K  E  M  A  T  I  F  L  F  A  L  L  A  A  G  F  T  S  S  A  M  A  A  S  G  S  D  S  G  P  W  D  F  D  I  Y  F  S  G
SL  ATGAAACGATTTAAAGAAATGGCAACAATTTTTCTTTTTGCATTGCTGGCTGCGGGGTTCACGTCGTCTGCTATGGCAGCGTCGGGTAGTGATAGT---CCGTGGGATTTTGAT------TTTTCGGGT
ST  ATGAAACGATTTAAAGAAATAGCAGCAATTTTGCTTTTTGCATTGCTGGCCGTGGGGTTCACGTCGTCGGCTATGGCATCGTCGGGCAGTGGTGGTGGCCCGTGGGATTTTGATATCTATTTTCCTGGT
SE  ATGAAACGATTTAAAGAAATGGCAACAATTTTTCTTTTTGCATTGCTGGCTGCGGGGTTCACGTCGTCTGCTATGGCAGCGTCGGGTAGTGATAGT---CCGTGGGATTTTGAT------TTTTCGGGT

    P  W  L  F  C  K  L  Q  P  P  D  S  L  Q  L  P  P  P  P  G  E  Y  C  W  H
SL  CCATGGCTTTTTTGCAAACTACAACCCCCTGATTCTCTGCAATTACCGCCACCTCCTGGTGAGTATTGCTGGCATTAA
ST  CACTACTTTTTTTGCAAACTACAACCCCCTGATTCTCTGCAATTACCGCCACCTCCTGGTGAGTATTGCTGGCATTAA
SE  CCATGGCTTTTTTGCAAACTACAACCCCCTGATTCTCTGCAATTACCGCCACCTCCTGGTGAGTATTGCTGGCATTAA
```

STsORF80

```
    M  K  E  G  F  Y  W  I  Q  H  N  G  R  V  Q  V  A  Y  Y  T  H  G  V  T  E  D  L  E  T  G  Q  T  I  I  G  V  W  H  L  T  Q  G  D
SL  ATGAAAGAAGGCTTCTACTGGATACAGCACAACGGCAGGGTTCAGGTTGCTTACTACACCCACGGCGTAACCGAGGACCTGGAAACTGGTCAGACTATTATTGGTGTCTGGCATCTGACGCAGGGCGAT
SE  ATGAAAGAAGGCTTCTACTGGATACAGCACAACGGCAGGGTTCAGGTTGCTTACTACACCCACGGCGTAACCGAGGACCTGGAAACTGGTCAGACTATTATTGGTGTCTGGCATCTGACGCAGGGCGAT
LA  ATGGAAGAAGGTTTCTACTGGATACAGCACAACGGCAGGGTTCAGGTTGCCTACTACACCAACGACGAGACCGAAGACCTGGAAACGGGCCGGATCATAACTGGTGTCTGGCATCTGACGCAGGGCGAT

    D  I  C  H  N  G  E  A  E  I  L  A  G  P  L  E  P  P  I
SL  GACATTTGTCACAACGGAGAGGCTGAGATTCTGGCGGGACCGTTAGAACCTCCAATTTAA
SE  GACATTTGTCACAACGGAGAGGCTGAGATTCTGGCGGGACCGTTAGAACCTCCAATTTAA
LA  GACATCTGCGATAACGGTGAGGCTGAGATTTTATCAGGACCGTTAACGCCGCCACATGCCTGA
```

STsORF99

```
    M  F  I  A  W  Y  W  I  I  L  I  V  L  V  V  V  G  Y  I  C  H  M  K  R  Y  C  R  A  F  R  Q  D  R  D  A  L  L  E  A  R  T  K  R
SL  ATGTTTATTGCCTGGTATTGGATAATATTAATCGTTCTGGTGGTAGTAGGGTATATTTGCCATATGAAGCGTTATTGCAGAGCGTTTCGGCAAGACAGAGATGCATTACTTGAAGCGCGAACGAAACGT
ST  ATGTTTATTGCCTGGTATTGGATAATATTAATCGTTCTGGTGGTAGTAGGGTATATTTGCCATATGAAGCGTTATTGCAGAGCGTTTCGGCAAGACAGAGATGCATTACTTGAAGCGCGAACGAAACGT
SE  ATGTTTATTGCCTGGTATTGGATAATATTAATCGTTCTGGTGGTAGTAGGGTATATTTGCCATATGAAGCGTTATTGCAGAGCGTTTCGGCAGGACAGAGATGCATTACTTGAAGCGCGAACGAAACGT
SG  ATGTTTATTGCCTGGTATTGGATAATATTAATCGTTCTGGTGGTAGTAGGGTATATTTGCCATATGAAGCGTTATTGCAGAGCGTTTCGGCAGGACAGAGATGCATTACTTGAAGCGCGAACGAAACGT
SB  ATGTTTATTGCCTGGTATTGGATCATATTCATTGTTCTTGTGGTAATAGGGTATATTTGCCATATGAAGCGTTATTGCAGAGCGTTTCGGCAGGACAGAGATGCATTACTTGAAGCGCGAAGAAAACTT

    F  R  Q  T  S  E  G  D  S  V  M  N  E  Q  K
SL  TTTCGCCAGACGAGCGAAGGCGACAGCGTGATGAATGAGCAGAAATAG
ST  TTTCGCCAGACGAGCGAAGGCGACAGCGTGATGAATGAGCAGAAATAG
SE  TTTCGCCAGACGAGCGAAGGCGACAGCGTGATGAATGAGCAGAAATAG
SG  TTTCGCCAGACGAGCGAAGGCGACAGCGTGATGAATGAGCAGAAATAG
SB  TTCCGCCAGATGAACGAAGGCGATAGCGCGATAAATGAGCAAAAATAG
```
