# Supplementary figures and images for: A global data-driven census of Salmonella small proteins and their potential functions in bacterial virulence
Source: Microlife. 2020 Oct 17;1(1):uqaa002. doi: 10.1093/femsml/uqaa002 (PMC10117436; doi:10.1093/femsml/uqaa002)

Figure S1

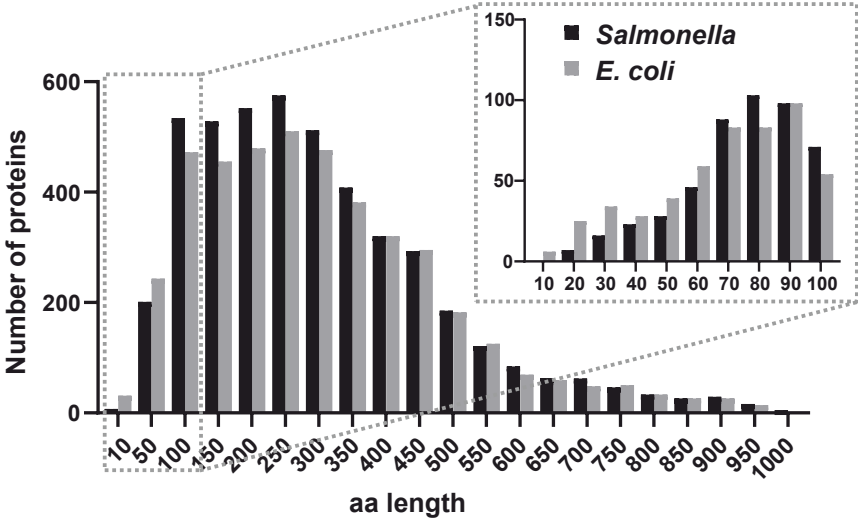

Supplement: uqaa002_Supplemental_Files [file uqaa002_supplemental_files.zip › Fig.S1.pdf]

Figure S4

a

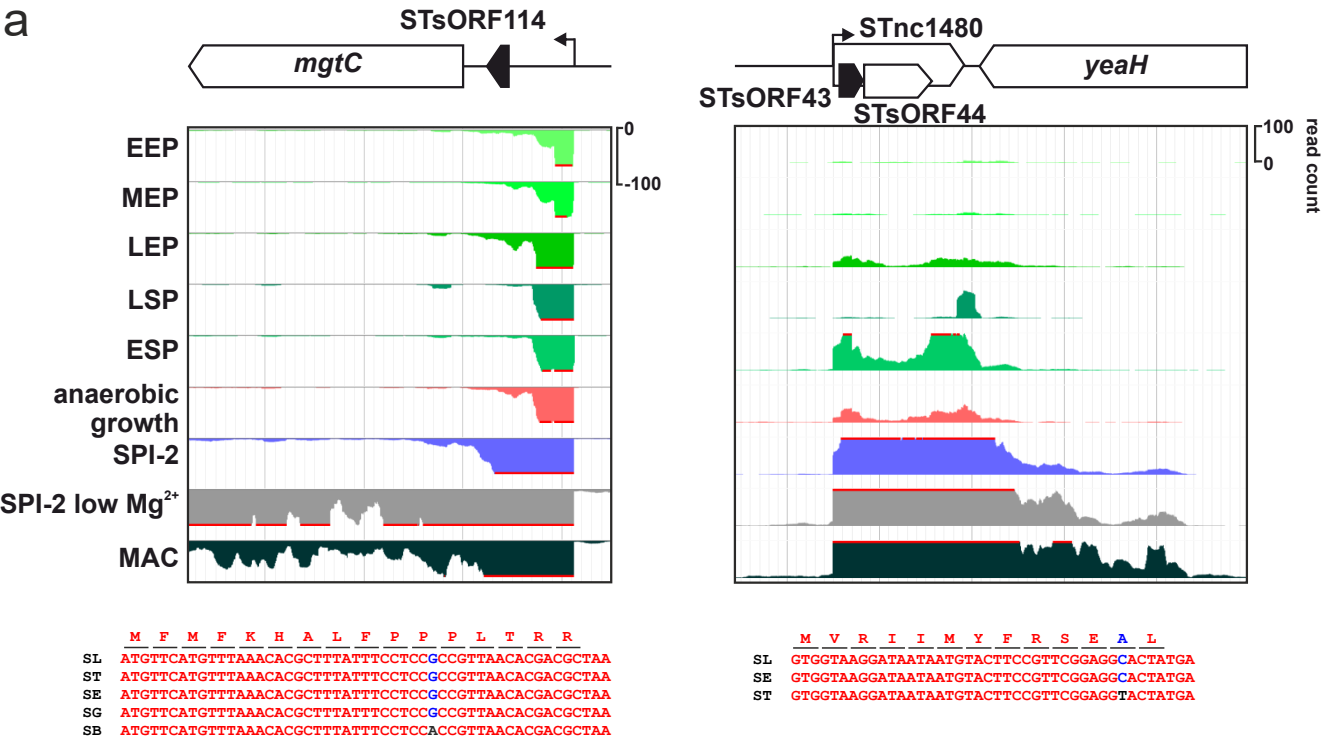

b

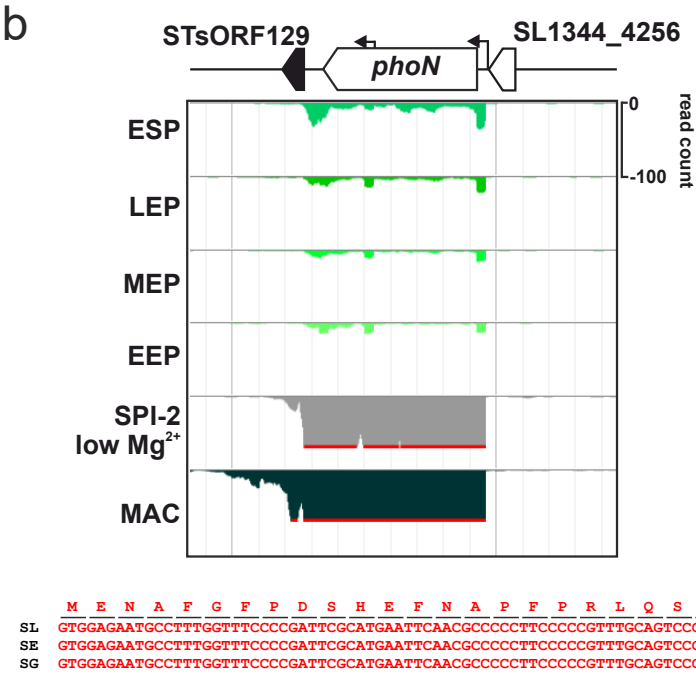

c

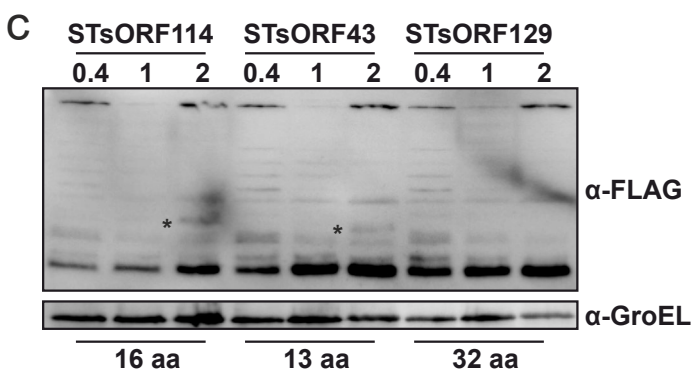

Supplement: uqaa002_Supplemental_Files [file uqaa002_supplemental_files.zip › Fig.S4.pdf]

Figure S5

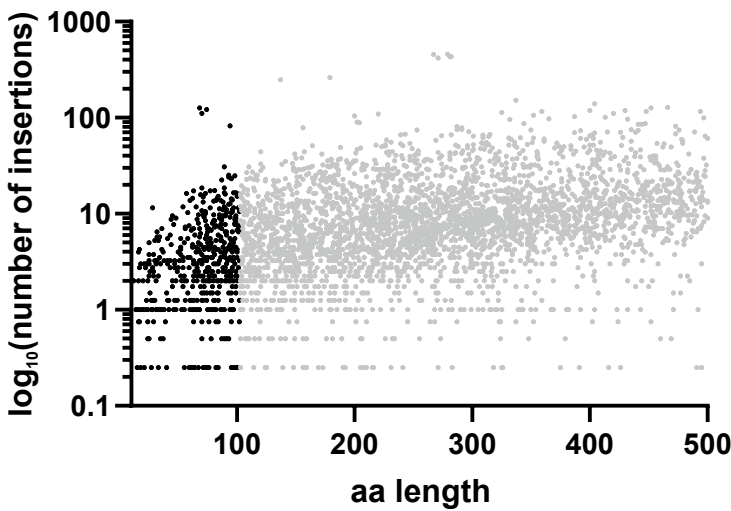

Supplement: uqaa002_Supplemental_Files [file uqaa002_supplemental_files.zip › Fig.S5.pdf]

Figure S6

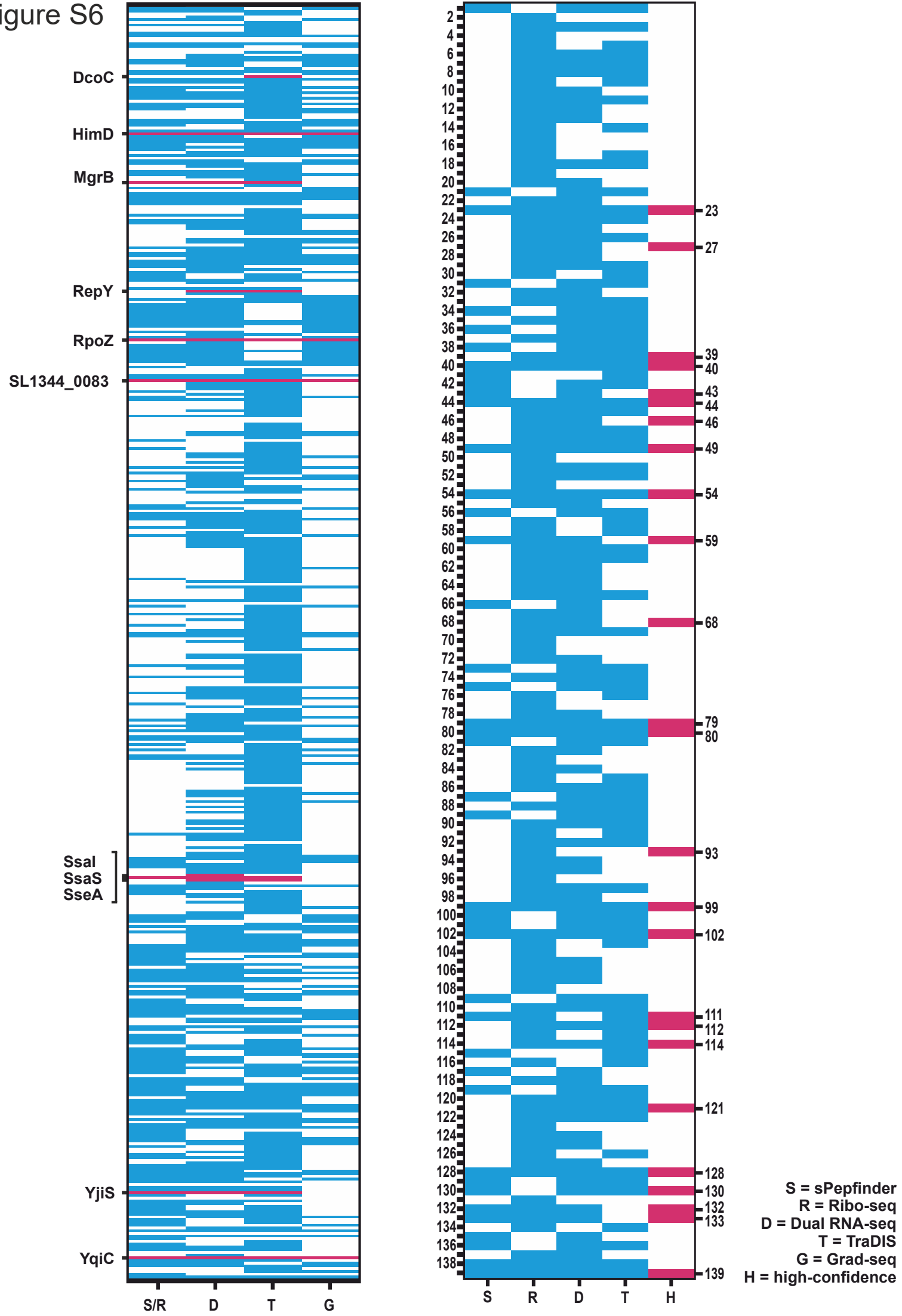

Supplement: uqaa002_Supplemental_Files [file uqaa002_supplemental_files.zip › Fig.S6.pdf]

Figure S7

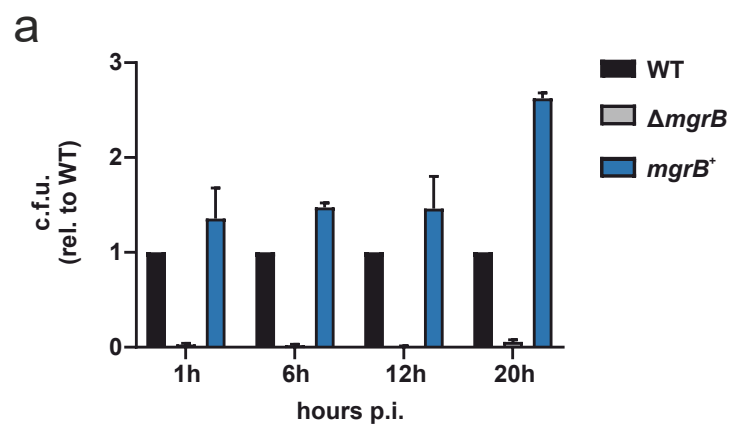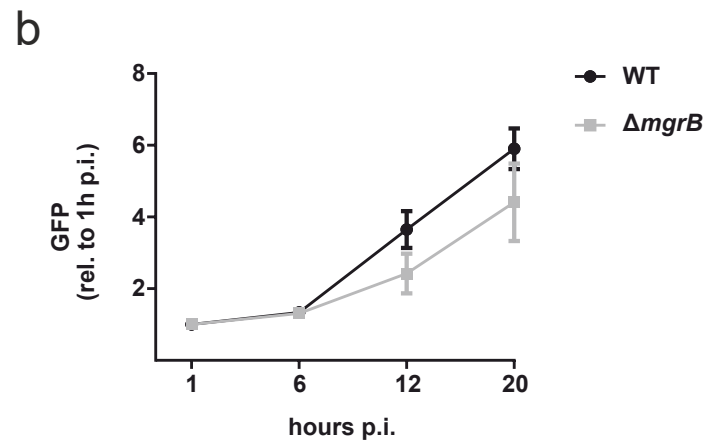

Supplement: uqaa002_Supplemental_Files [file uqaa002_supplemental_files.zip › Fig.S7.pdf]

Figure S8

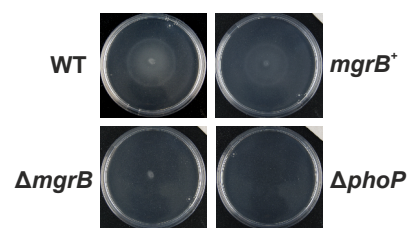

Supplement: uqaa002_Supplemental_Files [file uqaa002_supplemental_files.zip › Fig.S8.pdf]

Figure S9

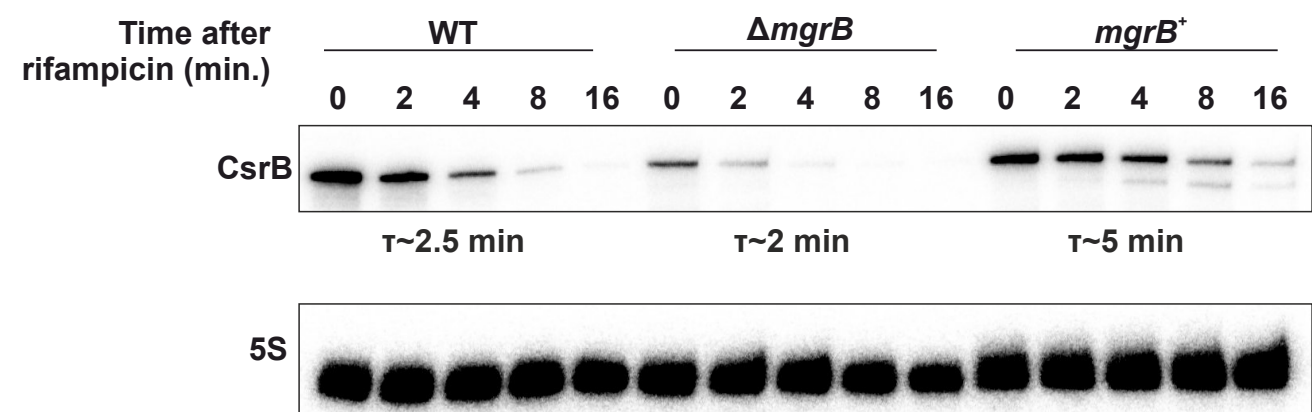

Supplement: uqaa002_Supplemental_Files [file uqaa002_supplemental_files.zip › Fig.S9.pdf]
